# Electrochemical Response of Glucose Oxidase Adsorbed on Laser-Induced Graphene

**DOI:** 10.3390/nano11081893

**Published:** 2021-07-23

**Authors:** Sónia O. Pereira, Nuno F. Santos, Alexandre F. Carvalho, António J. S. Fernandes, Florinda M. Costa

**Affiliations:** i3N, Department of Physics, University of Aveiro, 3810-193 Aveiro, Portugal; nfsantos@ua.pt (N.F.S.); alexandre.carvalho@ua.pt (A.F.C.); toze2@ua.pt (A.J.S.F.); flor@ua.pt (F.M.C.)

**Keywords:** Laser-Induced Graphene (LIG), electron transfer, Glucose Oxidase (GOx), flavin adenine dinucleotide (FAD), cyclic voltammetry

## Abstract

Carbon-based electrodes have demonstrated great promise as electrochemical transducers in the development of biosensors. More recently, laser-induced graphene (LIG), a graphene derivative, appears as a great candidate due to its superior electron transfer characteristics, high surface area and simplicity in its synthesis. The continuous interest in the development of cost-effective, more stable and reliable biosensors for glucose detection make them the most studied and explored within the academic and industry community. In this work, the electrochemistry of glucose oxidase (GOx) adsorbed on LIG electrodes is studied in detail. In addition to the well-known electroactivity of free flavin adenine dinucleotide (FAD), the cofactor of GOx, at the expected half-wave potential of −0.490 V vs. Ag/AgCl (1 M KCl), a new well-defined redox pair at 0.155 V is observed and shown to be related to LIG/GOx interaction. A systematic study was undertaken in order to understand the origin of this activity, including scan rate and pH dependence, along with glucose detection tests. Two protons and two electrons are involved in this reaction, which is shown to be sensitive to the concentration of glucose, restraining its origin to the electron transfer from FAD in the active site of GOx to the electrode via direct or mediated by quinone derivatives acting as mediators.

## 1. Introduction

Carbon-based materials (e.g., graphene, carbon nanotubes, etc.) are among the most promising electrodes for electrochemical transduction [1,2,3]. Graphene has been widely explored for biosensing platforms due to its outstanding physicochemical properties, namely the high surface area, excellent electric conductivity, swift electrochemical electron transfer and ease of surface functionalization [4,5,6,7]. Particularly, 3D porous graphene presents better mechanical stability and higher catalytic activity [8]. In 2014, Lin et al. [9] reported the synthesis of laser-induced graphene (LIG) on a polyimide sheet via direct laser scribing (DLS) processing. In this method, a 3D porous graphene foam is formed by a photothermal mechanism after laser irradiation on the substrate. LIG presents a high specific surface area, it is rapidly and cost-effectively produced with the desired pattern on a flexible substrate, attracting a great deal of attention in many fields [10,11]. Particularly, LIG appears as a candidate for the development of low-cost and flexible electrochemical biosensors [12,13]. For example, LIG decorated with copper nanoparticles (Cu NPs) was investigated for glucose detection by cyclic voltammetry (CV) and amperometric measurements [14,15]. In this non-enzymatic biosensor, Cu NPs promote the oxidation of glucose in alkaline media, and, under these conditions, the sensor does not respond to interferents such as ascorbic acid (AA), uric acid (UA), dopamine (DA), acetaminophen [14] neither other sugars (e.g., lactose, sucrose, fructose) [15]. As concerns the enzymatic biosensors, LIG modified with electrodeposited Pt NPs was explored to detect glucose by the amperometric response when H_2_O_2_, as a by-product of the enzymatic reaction, is oxidized by the Pt NPs [16,17]. Another example relies on the immobilization of Prussian blue, as electron mediator, on LIG electrodes to detect glucose oxidation by the enzyme [18]. LIG was also explored for the detection of the biomarkers AA, UA and DA [4,19,20], and for the sensing of thrombin [21] and urea [22] using specific biorecognition elements. Furthermore, LIG modified with molecularly imprinted polymers was used for detection of chloramphenicol, AA and amoxicillin [23,24].

*Diabetes Mellitus* is a disease affecting millions of people worldwide, thus research on glucose biosensors still has a high demand and impact on academia and industry pursuing cheaper, highly accurate, minimally or non-invasive methods to quantify glucose levels in real-time [25,26,27]. The first report of a biosensor dates back to 1962 by Clark and Lyons [28], for the amperometric detection of glucose by following the consumption of O_2_, which is a substrate of the enzymatic glucose oxidation.

**Scheme 1:**
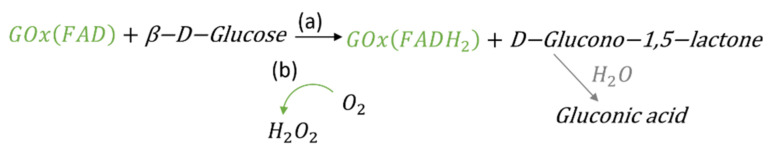
Representation of the enzymatic oxidation of glucose by the GOx enzyme in the presence of O_2_, evidencing the oxidized and reduced form of the cofactor FAD.

Enzymatic electrochemical glucose biosensors are the most explored, using the glucose oxidase (GOx) enzyme as biorecognition element adsorbed, trapped or covalently bonded to an electrochemical transducer. GOx is a homodimer, composed by two identical units, each one possessing in its active site the cofactor flavin adenine dinucleotide (FAD). The fully oxidized form of FAD catalyzes the oxidation of *β*-D-glucose (Scheme 1a) and then the GOx, with the reduced form of FAD (FADH_2_), is regenerated in the presence of O_2_ producing H_2_O_2_ (Scheme 1b) [29].

Three generations of biosensors have been developed based on this biochemical reaction [26,27]. In the first generation, glucose was quantified as a result of the enzyme activity, either via the consumption of O_2_, following the electrochemical reduction of O_2_ (Equation (1)), or through the oxidation of generated H_2_O_2_ (Equation (2)).

(1) $$O_{2}+4H^{+}+4e^{-}⟶\;2H_{2}O$$

(2) $$H_{2}O_{2}⟶\;2H^{+}+2e^{-}+O_{2}$$

In the second generation of glucose biosensors, known as mediated electron transfer (MET) biosensors, redox mediators are used to promote the electron transfer from FAD to the electrode’s surface. These electron-transfer mediators are needed because the FAD group is deeply buried in the active site of the enzyme, at least 17 Å from the enzyme surface [30,31]. In the oxidative half-reaction (Scheme 1b), O_2_ acts as the natural electron acceptor. In the MET mechanism, the mediators substitute or compete with O_2_ as electron acceptors allowing the regeneration of the reduced form of FAD in the active site of GOx, as well as the occurrence of electron transfer to the electrode surface [25,32]. The most used and studied redox mediators are ferrocene-derivatives, with formal potentials (E^0^) ranging from +0.14 to +0.44 V vs. Ag/AgCl [33,34,35], and for instance 1,4-benzoquinone with E^0^ approximately at +0.14 V vs. Ag/AgCl [36]. Usually, they are used freely in solution, but this hinders their application in vivo and potential toxicity risks exist [27]. Therefore, mediators immobilized on the enzyme surface have been explored, such as quinone derivatives [37,38], ferrocene derivatives [39] and gold nanoparticles [40,41] among others [42,43,44,45]. For more detail about GOx reengineering aiming to increase the rate of electron transfer to the electrode surface refer to [25].

Envisaging the development of mediator-free biosensors, a third generation has been explored, focusing on the direct electron transfer (DET) between the FAD in the active site of the enzyme (GOx(FAD)) and the surface of the electrode (without the need for mediators), following Equation (3).

(3) $$GOx(\mathrm{FAD})+2e^{-}+2H^{+}\overset{\left(1\right)}{\underset{\left(2\right)}{⇌}}GOx(\mathrm{FAD}H_{2})$$

Hundreds of works have been published reporting DET and the use of this mechanism for the detection of glucose [31]. However, in 2013 [36] and 2014 [46], different groups clarified that DET does not occur, and the typical pair of anodic and cathodic peaks observed, at circa −0.45 V vs. Ag/AgCl (3 M KCl) at pH 7, are due to the electroactivity of free FAD adsorbed by the electrodes, i.e., FAD that is not part of the active site of the enzyme. In fact, in 2016, an editorial note from G. S. Wilson on Biosensors and Bioelectronics [47] states that DET does not occur. This was also demonstrated in 2018 [31] and, since then, it has become well established in the scientific community that the effect of DET has not yet been observed. The misleading analysis of DET arises from the free FAD as an impurity of GOx powder or due to FAD extrusion during GOx adsorption on the electrode. In addition, the cathodic peak is overlapped with the reduction potential of O_2_ (described in Equation (1)) thus, when in the presence of glucose, its catalytic oxidation leading to consumption of O_2_, and a decrease in peak current is observed. Therefore, this corresponds to a first-generation biosensor mechanism, instead of a third generation one.

Given the general interest on LIG for biosensing purposes and glucose detection/quantification in particular, the electrochemistry of GOx adsorbed on LIG is studied in this work. In addition to the well-known free FAD electroactivity at ca. −0.490 V vs. Ag/AgCl (1 M KCl), a second pair of peaks at +0.155 V vs. Ag/AgCl (1 M KCl) is reported for the first time after the adsorption of GOx on the LIG electrodes. Scan rate and pH, along with a set of tests in the presence of glucose were performed in order to determine the origin of such peaks. A discussion about the observation of this new faradaic activity of LIG/GOx and their possible relationship with electron transfer from GOx to the electrode surface, via either a direct or mediated mechanism, is presented.

## 2. Materials and Methods

### 2.1. Reagents

Glucose oxidase (GOx) from *Aspergillus niger* (Type VII, lyophilized powder, ≥100,000 units/g solid), D-(+)-Glucose (≥99.5% (GC), powder), flavin adenine dinucleotide (FAD) disodium salt hydrate (≥95% (HPLC), powder), sodium phosphate monobasic monohydrate (NaH_2_PO_4_.H_2_O, ACS reagent, ≥98%), sodium phosphate dibasic heptahydrate (Na_2_HPO_4_.7H_2_O, ≥99.99% trace metals basis) and cholesterol oxidase (ChOx) from Streptomyces sp. (lyophilized powder, ≥20 units/mg protein) were purchased from Sigma-Aldrich, Munich, Germany. Phosphate Buffer Saline (PBS) tablets (pH = 7.4, 10 mM of phosphate ions, 0.137 M NaCl and 0.027 M KCl) were purchased from Fisher Bioreagent, Massachusetts, USA. Deionized (DI) water was obtained from a MilliQ water purification system. All reagents were used as received.

### 2.2. LIG Synthesis and Electrodes Fabrication

**Figure 1:**
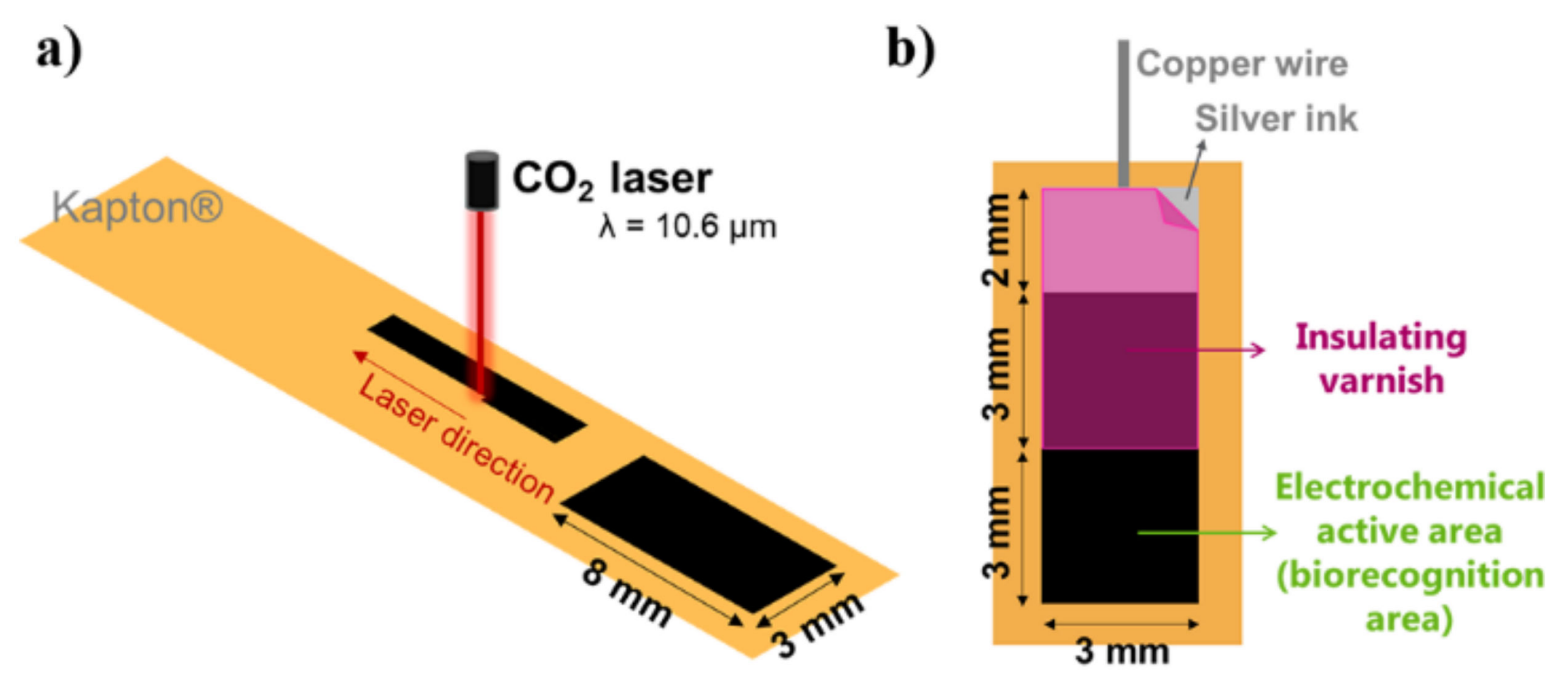
Schematic representation of (**a**) LIG synthesis by the DLS method and (**b**) LIG electrode produced for electrochemical measurements.

Laser-induced graphene (LIG) was synthesized by a direct-laser scribing (DLS) method (Figure 1). A polyimide sheet (Kapton^®^ NH300 with 75 μm in thickness, DuPont, Wilmington, Delaware, USA) was irradiated with a continuous CO_2_ laser (Redsail M500, Jinan, Shandong Province, China) with a wavelength of 10.6 μm, a maximum power output of 50 W and a beam diameter of circa 100 μm. This system is fitted with a gantry driven X-Y focusing head. In this work, the optimized laser parameters were chosen based on previous work [4], where a detailed morphological, structural and electrochemical characterization was performed. The distance between laser lines (*d*) was 0.075 mm, the laser power output was ~12.5 W, the laser scan speed *ν_laser_* was 250 mm s^−1^ and the distance between the laser head and the substrate *d_laser_* was 1.98 cm (note that the focused condition is *d_laser_* = 1.80 cm). Kapton^®^ sheets were cleaned with ethanol before processing and LIG was synthesized under ambient conditions.

LIG was produced with a geometrical area of 3 × 8 mm^2^ as represented in Figure 1a and cleaned with DI water. Afterwards, an electrical contact was established using a copper wire and silver ink (Electrodag 1415 from Agar Scientific, Essex, UK) baked at 120 °C for 20 min. The electrical contact was covered with an insulating varnish (Lacomit from Agar Scientific, Essex, UK) that also aids in defining the geometric electroactive area (3 × 3 mm^2^) of the electrode, as represented in Figure 1b).

### 2.3. FAD and GOx Adsorption on LIG Electrodes

FAD and GOx were immobilized on the LIG electrode by physical adsorption, following the same procedure. A fresh solution of the enzyme (5 mg mL^−1^) was prepared in PBS and the electrode was immersed in 500 μL overnight, and kept refrigerated at ~10 ºC. Afterwards, electrodes were exhaustively washed with PBS to remove non-adsorbed enzyme. In the case of FAD, a solution of 10 mM was used.

Following a similar procedure, Cholesterol oxidase (ChOx) enzyme was also immobilized on LIG electrodes by physical adsorption, see the detailed description in Supplementary Materials.

### 2.4. Morphological and Structural Characterization

Micro-Raman spectroscopy was carried out in a Horiba HR800 instrument (Horiba Scientific, Kyoto, Japan) in the backscattering configuration by exciting the LIG with a 442 nm line from a CW He-Cd laser (Kimmon IK Series, Fukushima, Japan), using a ND 0.6 neutral density filter and focusing with a 50× magnification objective with a numerical aperture of 0.7. Scanning electron microscopy (SEM) was performed with a TESCAN VEGA3 microscope (TESCAN, Libušina, Czech Republic) in the secondary electron acquisition mode (SE). The samples were analyzed high-resolution transmission electron microscopy (HR-TEM) using a Field Emission Microscope Jeol JEM-2200FS (JEOL, Tokio, Japan) operated in imaging mode, at 200 keV. A drop of LIG dispersed in ethanol was placed in a holey carbon film copper grid and left to evaporate in air. XPS spectra were acquired in an ultra-high vacuum system with a base pressure of 2 × 10^−10^ mbar. The system was equipped with a hemispherical electron energy analyzer (SPECS Phoibos 150, SPECS, Berlin, Germany), a delay-line detector and a monochromatic AlKα (1486.74 eV) X-ray source. High resolution spectra were recorded at normal emission take-off angle and with a pass-energy of 20 eV, which provides an overall instrumental peak broadening of 0.5 eV. The XPS data were fitted using the free software XPSPEAK 4.1, a Shirley background was applied, and for sp^2^ carbon, an asymmetric peak shape model was used, as is typically employed for graphene.

### 2.5. Electrochemical Measurements

Electrochemical characterization was performed using a VersaSTAT3 electrochemical station (Princeton Applied Research, USA) in a home-made three-electrode configuration setup where LIG was the working electrode (WE, geometric area 0.09 cm^2^), Ag/AgCl (1 M KCl) (CHI111, CH Instruments, Inc, Texas, USA) the reference electrode (RE), and a Pt wire the counter electrode (CE), see Figure S2 in Supplementary Materials. The home-made electrochemical cell is a closed system with an inlet for N_2_ allowing the purging of O_2_ (the measurements in the presence or absence of O_2_ are indicated throughout the work). All the measurements were performed in 35 mL of PBS (pH 7.4, 10 mM) as electrolyte at room temperature, except in the case of the pH- and temperature- dependence study.

#### 2.5.1. LIG Activation/Stabilization

LIG was submitted to a conditioning procedure in order to stabilize the electrochemical response before being used throughout the work. To do so, all the LIG electrodes were immersed in PBS for ~30 min and then 10 voltammogram cycles (2 × 5 cycles) were run from −1.0 to 1.0 V at 100 mV s^−1^, using PBS (pH 7.4, 10 mM) as the electrolyte. See the CVs in detail in Figure S3 in Supplementary Materials.

#### 2.5.2. pH-Dependence Study

All the solutions were prepared keeping the ionic strength constant (0.137 M NaCl and 0.027 M KCl): for pH 5.8 a mixture of NaH_2_PO_4_.H_2_O (9.5 mM) and Na_2_HPO_4_.7H_2_O (0.5 mM) was used; for pH 4.7 and 2.8, NaH_2_PO_4_.H_2_O (10 mM) was used and the pH was adjusted using HCl; for pH 8.8 and 10.3, Na_2_HPO_4_.7H_2_O (10 mM) was used and the pH was adjusted using NaOH.

#### 2.5.3. Glucose Detection Tests

A stock solution of glucose was prepared in PBS with a concentration of 500 mM. The electrolyte (35 mL of PBS) was bubbled for 30 min in order to remove O_2_ and five cycles of CV were recorded. Then, small volumes of the glucose stock solution (30, 60, 150, 300, 600 and 1200 μL) were added during stirring under a flux of N_2_ to guarantee the absence of O_2_ in the electrolyte. After each volume addition, the stirring was stopped, and three CVs were run. For the glucose detection test in the presence of O_2_, the N_2_ bubbling did not occur.

## 3. Results

### 3.1. Morphological and Structural Characterization of LIG

**Figure 2:**
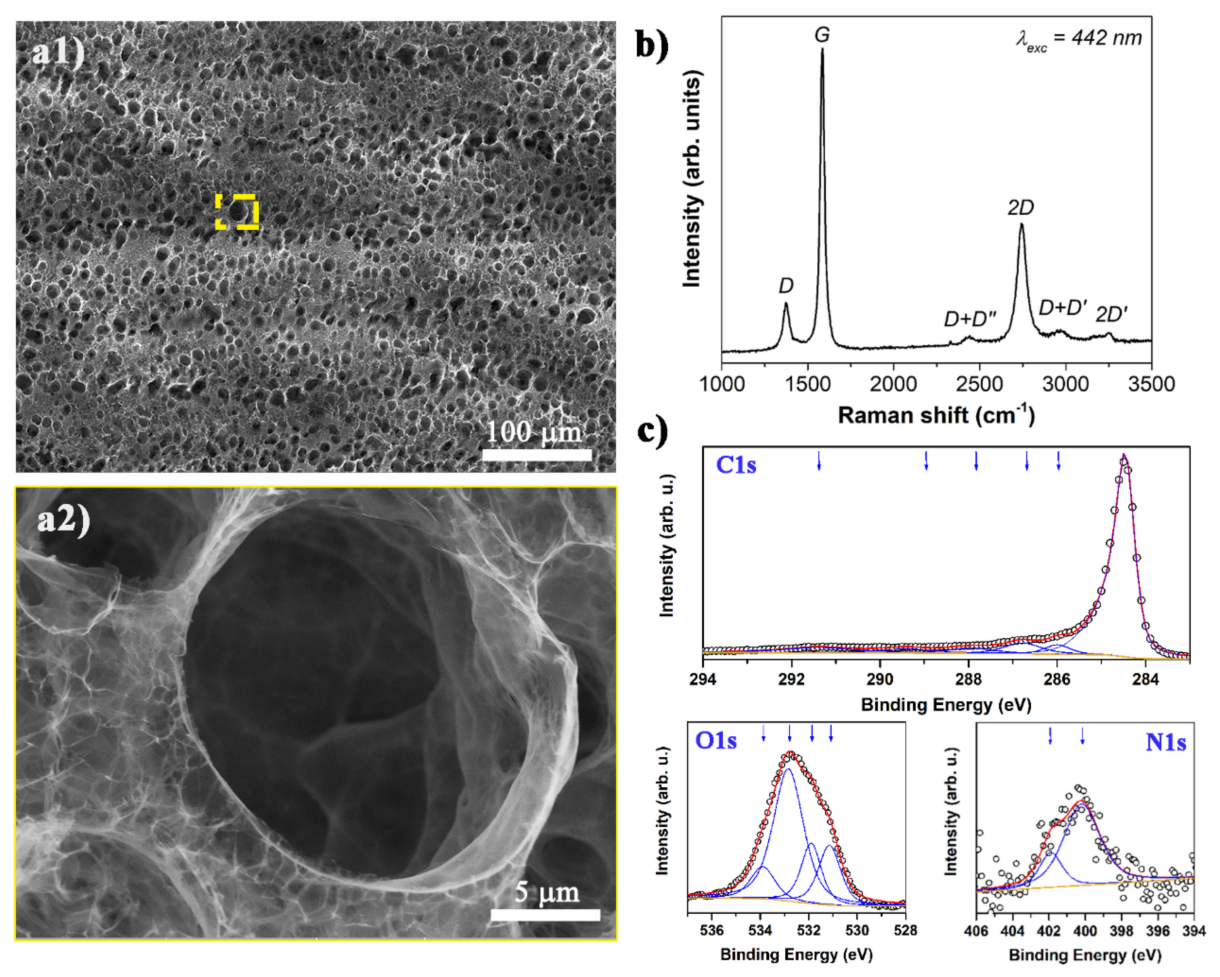
LIG surface characterization. (**a1**) SEM image of LIG; (**a2**) High magnification SEM image of a pore in image (**a1**) highlighted with a dash red line; (**b**) Raman spectrum using 442 nm laser excitation wavelength; and (**c**) XPS spectra of C1s, O1s e N1s regions, red, blue and yellow lines refers to the original spectra, the deconvoluted peaks and the baseline used, respectively.

The low magnification SEM image (Figure 2a1) shows the morphological structure of LIG, where it is possible to observe the laser path scan forming “valleys” and “hills”, in which the latter correspond to the overlapped regions of the laser scan. Noticeably, LIG has an edgy and porous structure, with visible pores with a hierarchical distribution from 3 to 20 μm in diameter. A close inspection at higher magnification (Figure 2a2) reveals smaller pores inside the bigger ones, suggesting an intricate pore network interesting for enhanced enzyme loading. The high density of edges is also interesting in terms of added electrochemical activity [19].

A representative Raman spectrum of the LIG samples is presented in Figure 2b. The fingerprint vibrational modes of graphene-based materials are identified, namely the G, D and 2D bands at 1586 cm^−1^, 1372 cm^−1^ and 2742 cm^−1^, respectively. The D band arises from defect-activated breathing modes of the hexagonal sp^2^ carbon structure, while the G band is related to the C–C bond stretching mode in sp^2^ phases. The ratio of intensities between D and G bands (I_D_/I_G_) is 0.15, indicating a considerable density of defects on the graphene structure, as would be expected [4,9,48] taking into account the LIG morphology, as previously seen in the SEM imaging. However, an intense D band alongside with an imperceptible D’ band suggests that the defects are mostly due to presence of sp^3^-linked functional groups, and not by the effect of the edges [49]. The second order D band overtone, the 2D band, is well defined and the I_2D_/I_G_ ratio is about 0.4, indicative of multi-layered graphene [48]. Moreover, it is symmetric showing that no apparent stacking ordering of the graphene layers exists. This indicates that LIG is formed by the incommensurate stacking of graphene layers [4,50]. Finally, well defined D+D’’, D+D’ and 2D’ overtones are also present, a common feature in the Raman spectra of good structural quality graphene [48]. The multilayer graphene structure of LIG is also observable in the TEM image of Figure S1 in Supplementary Materials, showing an interlayer spacing of 3.6 Å, in agreement with the reported elsewhere [4].

High resolution X-ray photoelectron spectroscopy (XPS) was performed to understand the chemical nature of the 3D porous graphene produced by DLS. Three elements were identified: C, O and N in a ratio 83.4/16.0/0.6, and the corresponding XPS spectra in the C1s, O1s and N1s regions are presented in Figure 2c. For carbon, several species were found at the following binding energies (BE): C1s: 284.5 eV (C=C, sp^2^), 286.0 eV (C – OH/C – N), 286.8 eV (C – O), 287.9 eV (C=O), 289.4 eV (O – C=O) and 291.3 eV (π–π satellite). For oxygen species, the following BE were assigned to: 531.1 eV (aliphatic O=C), 531.9 eV (aromatic O=C), 532.8 eV (C-O-C) and 533.9 eV (C-OH). Finally, LIG also revealed nitrogen in its chemical structure due to the polymer precursor (polyimide): 400.2 eV (pyrrolic N) and 401.9 eV (quaternary N). The pyridic N, at circa 398 eV, was not possible to identify in this fitting due to the background noise, although it has been reported in LIG materials [4,9,22,51]. The presence of these groups reinforces the suggested origin of the lattice defects probed by Raman and confirms that LIG has structural characteristics similar to reduced graphene oxide.

### 3.2. Electrochemical Measurements

**Figure 3:**
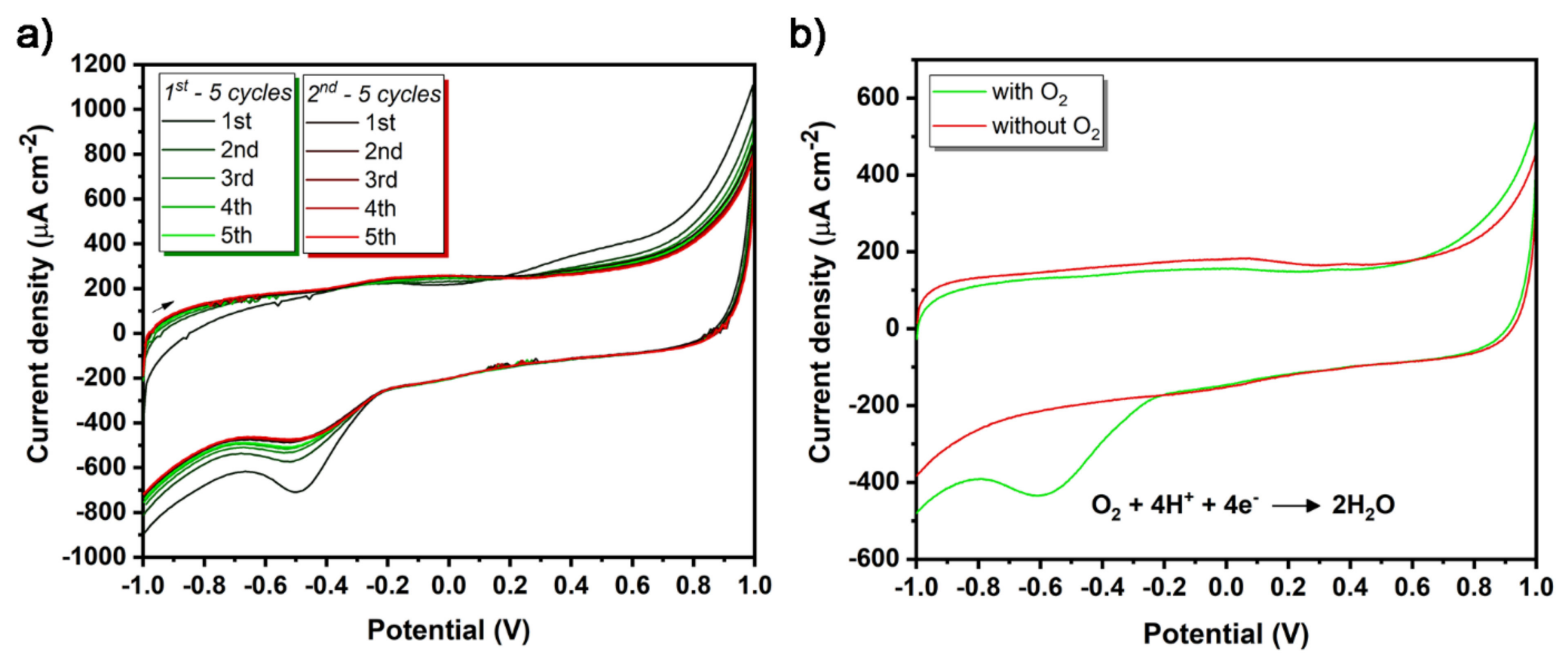
Cyclic voltammograms, from −1.0 to 1.0 V at 100 mV s^−1^, of LIG electrodes. (**a**) Running 10 cycles (2 × 5cycles) to stabilize the electrode before being used. (**b**) CVs after stabilization in the presence and absence of O_2_. PBS (pH 7.4, 10 mM) was used as electrolyte and the potentials were measured against Ag/AgCl (1 M KCl).

Prior to use, all the LIG electrodes were submitted to a conditioning procedure in order to stabilize the electrochemical background, as displayed in Figure 3a. Furthermore, a comparison of the CV of LIG electrodes in the absence and presence of O_2_ was conducted, see Figure 3b, where the peak corresponding to the reduction of oxygen, according to Equation (1), is clearly identified. Moreover, no additional faradaic activity was observable in the bare LIG electrodes.

As previously mentioned, most prior works erroneously describe DET from GOx to the electrode’s surface, as the electroactivity of free FAD and O_2_ are overlapped. In the following sections, the electrochemical analysis of FAD and GOx adsorbed on LIG electrodes is presented and discussed.

#### 3.2.1. FAD Adsorbed on LIG

**Figure 4:**
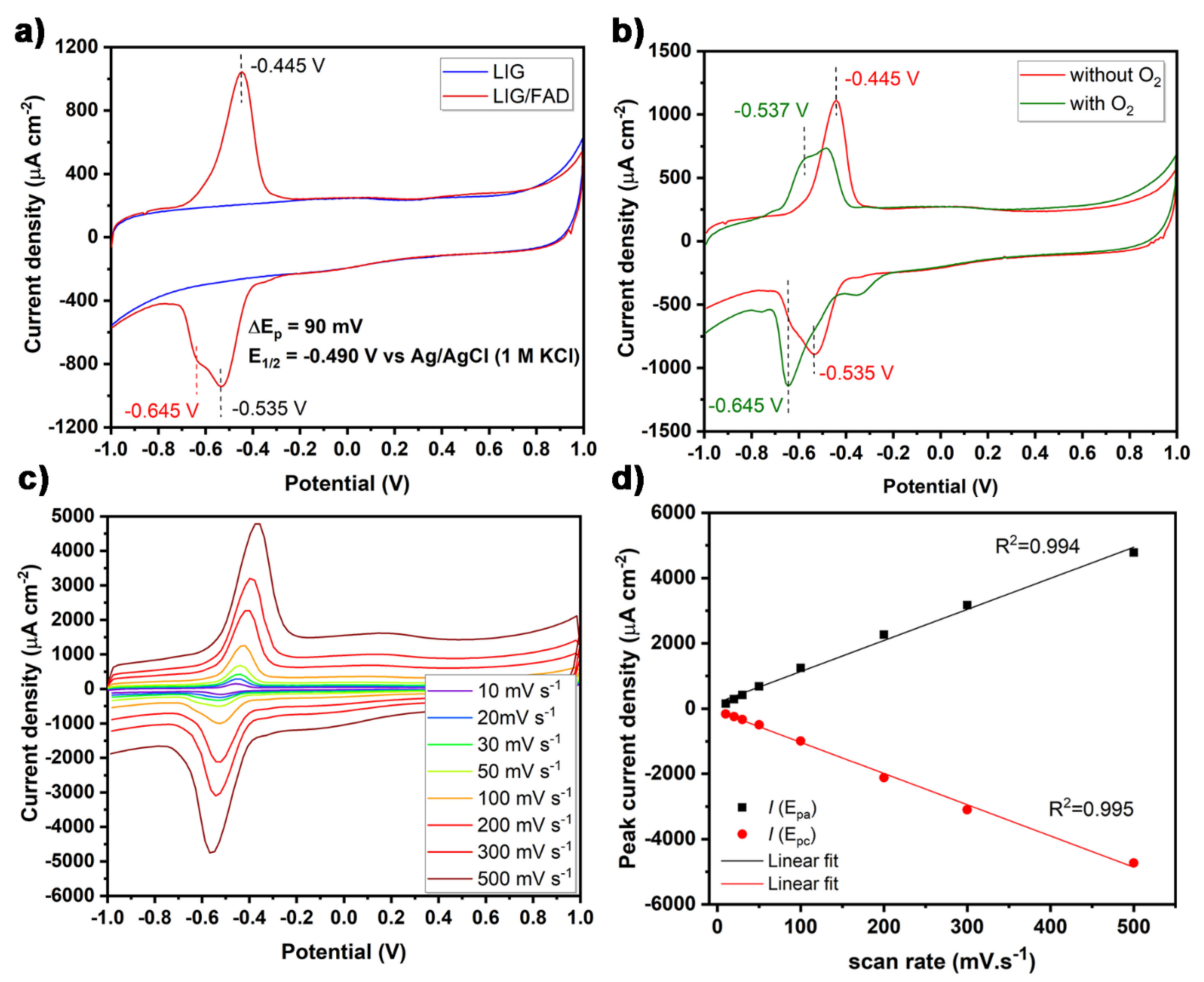
Cyclic voltammograms of (**a**) LIG before and after FAD adsorption, without O_2_, and (**b**) of LIG/FAD electrodes in the presence and absence of O_2_. (**c**) Cyclic voltammograms at different scan rates for the LIG/FAD electrode and (**d**) the respective intensity of current for anodic, *I*(E_pa_), and cathodic, *I*(E_pc_), peaks as a function of the scan rate.

**Scheme 2:**
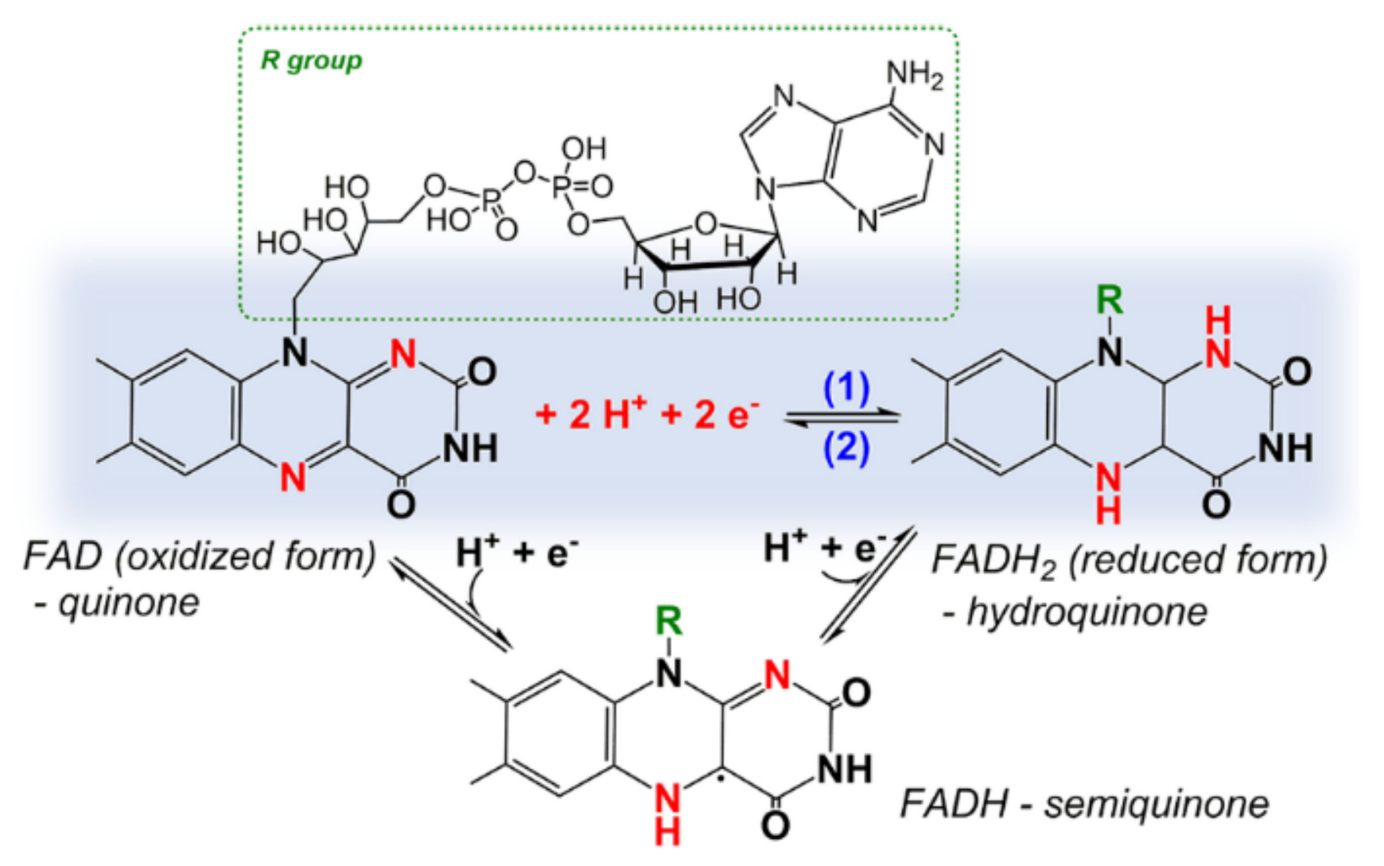
FAD chemical structure and schematization of redox FAD reaction via the two-electron mechanism (quinone to hydroquinone, highlighted in blue) or the one-electron mechanism (semiquinone intermediary formation).

It is well known that FAD strongly adsorbs on carbon-based electrodes, for example on graphite [52], glassy carbon [53], carbon nanotubes (CNT) and nitrogen-doped CNTs [54] following a Langmuir adsorption isotherm model. In LIG, FAD is also adsorbed as can be clearly observed in Figure 4 by the presence of a well-defined pair of anodic and cathodic peaks (E_pa_ and E_pc_, respectively) at −0.445 V and −0.535 V. The cathodic peak corresponds to the reduction of FAD (see Equation (1) in Scheme 2), while the anodic peak is ascribed to the oxidation of FADH_2_ (see Equation (2) in Scheme 2). This pair of peaks, at a scan rate of 100 mV s^−1^ and pH = 7.4, has a half-wave potential (E_1/2_) of −0.490 V vs. Ag/AgCl (1 M KCl), a peak-to-peak separation (ΔE_p_ = E_pa_−E_pc_) of 90 mV, and a similar current intensity for both peaks, which is in agreement with related literature [31,53,54].

Moreover, the cathodic peak presents a shoulder which is attributed to the formation of the semiquinone intermediate [53,55], i.e., when the reduction of FAD goes through the one-electron transfer mechanism, instead of the two-electron transfer as depicted in Scheme 2. Interestingly, the one-electron redox reaction seems to be favored in the presence of oxygen, as observed in Figure 4b, thus shifting the half-wave potential to more negative values. Furthermore, as observed in Figure 3, the cathodic peak between −0.7 and −0.4 V is ascribed to the reduction of O_2_, which is overlapped with the FAD reduction peak.

In the absence of O_2_, the anodic (*I*_Epa_) and cathodic (*I*_Epc_) peak currents denote a linear dependence on the scan rate from 10 to 500 mV s^−1^ (see Figure 4c,d). This indicates that the redox species (FAD/FADH_2_) are adsorbed at the surface, as expected, thus these reactions are surface-controlled [53]. Furthermore, using the data from the scan rate dependent CVs, the apparent electron transfer coefficient (*α*_app_) and heterogeneous electron transfer rate constant (*k*_app_) were calculated [53,56] using Equation (4):

(4) $$Ep-E_{1/2}=\frac{RT}{\alpha_{\mathrm{app}}nF}\ln\left(\frac{RTk_{\mathrm{app}}}{\alpha_{\mathrm{app}}nFv}\right)$$

where *R* is the gas constant (J mol^−1^ K^−1^), T is the temperature (K), *F* is the Faraday constant (C mol^−1^) and *ν* is the scan rate (Vs^−1^). A theoretical number of electrons involved in the reduction reaction (*n* = 2) was used. The results, *α*_app_ = 0.34 and *k*_app_ = 1.03 s^−1^, are in line with other works as summarized in [53]. Moreover, a concentration of FAD adsorbed on LIG (*Γ* = 1.3 × 10^−8^ mol cm^−2^) was estimated following the well-known Faradaic equation, where the integrated area of the cathodic peak is equal to *F* × *A* × *Γ* × *ν* (where *A* is the geometrical area of the electrode, 0.09 cm^2^). This value is slightly higher comparing with FAD adsorbed on glassy carbon electrodes (GCE) at neutral pH (*Γ* = 2.6 × 10^−10^ mol cm^−2^) [53] and comparable with FAD adsorbed on graphite, *Γ* = 2 × 10^−8^ mol cm^−2^, at acidic pH [52].

#### 3.2.2. GOx Immobilized on LIG Electrodes

**Figure 5:**
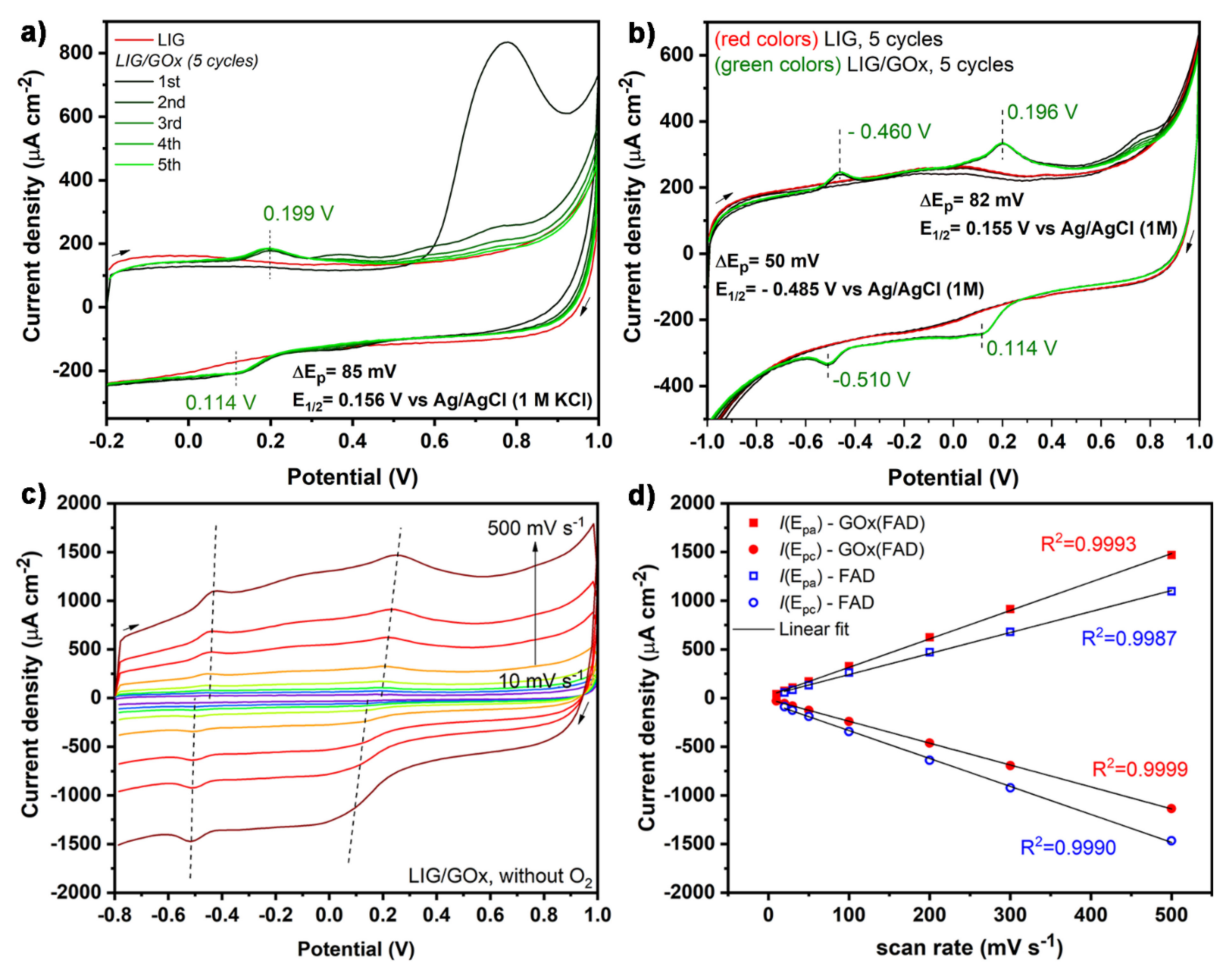
Cyclic voltammograms of: (**a**) LIG (the fifth cycle is presented) and LIG/GOx (five cycles are displayed) in a potential range from −0.2 to +1.0 V at 100 mVs^−1^; (**b**) LIG and LIG/GOx in a potential range from −1.0 to +1.0 V at 100 mVs^−1^ after CV in (a). (**c**) Cyclic voltammograms of the LIG/GOx electrode at different scan rates: 10, 20, 30, 50, 100, 200, 300 and 500 mVs^−1^; and (**d**) the respective anodic, *I*(E_pa_), and cathodic, *I*(E_pc_), peak currents of FAD and GOx(FAD) adsorbed on LIG, as a function of the scan rate. All the measurements were performed in the absence of O_2_.

GOx was immobilized on LIG electrodes via physical adsorption and then electrochemical measurements were conducted, as displayed in Figure 5. First, a CV was performed from −0.2 to 0.6 V (see Figure S3 in Supplementary Materials) but no faradaic activity was observed. After running the CV up to 1.0 V, an unpaired peak at +0.77 V is observed in the first cycle and its current decreases for the following cycles, while a new pair of peaks (E_1/2_ = +0.156 V in Figure 5a) appears after the first cycle and is always observed after that (see also Figure S4). The electrochemical nature of the peak at +0.77 V is not clear but it must be related to the presence of the enzyme on the LIG electrodes as it is only observed after GOx adsorption. It is worth remembering that the bare LIG electrodes were previously swept from −1.0 to +1.0 V (10 cycles) in order to stabilize the electrochemical background and the just-mentioned peaks were not observed, see the CV in Figure 3. In addition, the stabilized LIG electrode was used not only to adsorb the GOx but also the FAD as previously discussed in Section 3.2.1. In the FAD-adsorbed LIG electrode, neither the peak at +0.77 V nor the pair of peaks with E_1/2_ = +0.156 V were observed when the CV was run from −1.0 to +1.0 V. Therefore, the nature of these peaks cannot be related to electrochemical active groups from LIG nor from FAD, but arise from the electrochemical interaction between the GOx and LIG.

Thereafter, the CV of the LIG/GOx electrodes was run from −1.0 to +1.0 V, as demonstrated in Figure 5b, and two pairs of peaks were observed. The first one (E_1/2_ = −0.485 V, ΔE_p_ = 50 mV) has already been reported [46,57] and corroborated in the present work in the previous Section 3.2.1. It is ascribed to the electroactivity of adsorbed free FAD and is not a result of enzyme-active FAD, as discussed elsewhere [31,36]. Concerning the second pair of peaks (E_1/2_ = +0.155 V, ΔE_p_ = 82 mV), the following electrochemical measurements were performed to understand their nature. It is important to keep in mind that this pair of peaks only appears after running the CV up to +1.0 V, meaning that some electrochemical reaction occurred between the enzyme and LIG.

A scan rate study, from 10 to 500 mV s^−1^, was conducted for the LIG/GOx electrode in the absence of O_2_, see Figure 5c. In this scan rate range, both peak currents increase linearly with the increase in the scan rate, again meaning that the redox species are adsorbed, as presented in Figure 5d.

**Figure 6:**
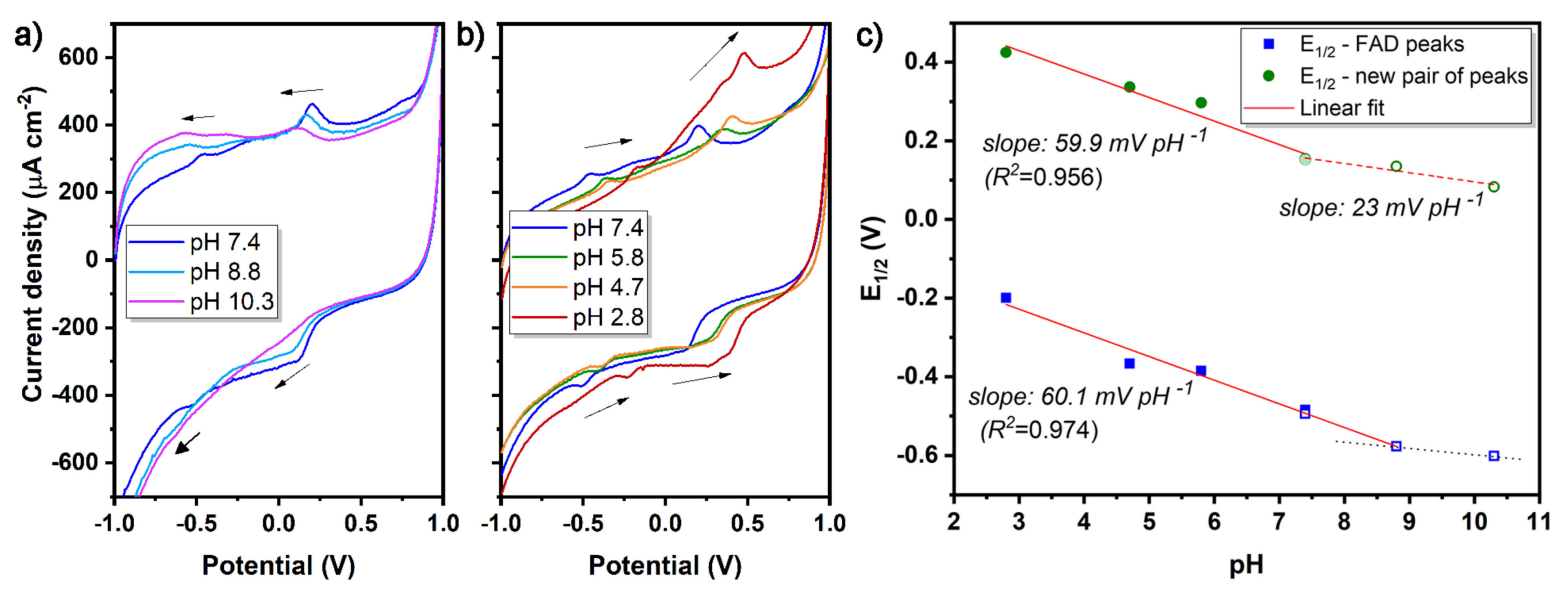
Cyclic voltamograms (100 mV s^−1^) of LIG/GOx varying the pH from (**a**) pH 7.4 to 10.3 and from (**b**) pH 7.4 to 2.8. (**c**) Half-wave potential (E_1/2_) of the new pair of peaks and the one corresponding to free FAD in function of the pH. In (**c**) the open and the closed symbols correspond to the data from (**a**) and (**b**), respectively. The dotted black line aids in guiding the eye to observe the change in the slope. All the measurements were performed in phosphate containing solutions with the same ionic strength (NaCl 0.137 M and KCl 0.027 M), in the absence of O_2_.

The cyclic voltammetry measurements of the LIG/GOx electrode in electrolytes with different pH values is displayed in Figure 6. It is well-known that the position of the anodic and cathodic peaks of FAD varies with pH since the redox reaction is proton-dependent [53,58]. For higher (alkaline) pH values, the pair of peaks corresponding to FAD shift to more negative potentials (at pH 7.4 the E_1/2_ is −0.49 V and at pH 10.3 it is −0.60 V), whereas for lower (acidic) pH values, the peak potentials shift to less negative ones (from pH 7.4 to 2.8, the E_1/2_ changes from −0.49 V to −0.20 V). Moreover, a slope of 60.1 mV pH^−1^, in the pH range from 3 to 8, was obtained plotting the E_1/2_ against pH, as depicted in Figure 6c. A value of *n* = 1.95 was calculated following the Nernst equation [53], which is very close to the expected theoretical value of *n* = 2 for the reaction involving two protons and two electrons (see Scheme 2). These values are in agreement with the literature for the pH dependence of FAD. For instance, using glassy carbon electrodes, a slope of 66.7 mV pH^−1^ was obtained, thus *n* = 1.76, meaning that a small portion of FAD goes through the one-electron redox process (semiquinone formation) [53]. Above pH 8, the slope seems to decrease indicating that the reaction mechanism changes, meaning that after this value the reaction follows a two electron and one proton mechanism by semiquinone formation (Scheme 2). Indeed, the free FAD in solution has a pKa between 6.4 and 6.8 [58], but for instance, after adsorption on graphite [54] the pKa shifts to a value above 8. Likewise, in this work, FAD adsorbed on LIG also shifts its pKa for a value above 8, showing the occurrence of a strong interaction between FAD and LIG.

Interestingly, when attention is focused on the second pair of peaks (E_1/2_ = +0.155 V), a similar pH-dependent behavior can be observed. For the lowest pH value, the peak seems to be composed by two overlapping peaks. At pH 10.3 the anodic peak is broader, resembling that of FAD at this pH [53], as above the pKa only one-proton is involved in the reaction instead of the two protons represented in Scheme 2. However, this is not clearly noticeable for free FAD since the peak is very weak and becomes weaker with increasing pH, as expected. Concerning the variation of the E_1/2_ against pH, a slope of 59.9 mV pH^−1^ was determined for the new pair of peaks, also corresponding to *n* = 1.95 following the Nernst equation [53]. In this case, the linear range is obtained below pH 8, indicating that in this case the pKa is closer to 8, more specifically between 7.4 and 8. In fact, for pH values above pH 7.4 a slope of 23 mV pH^−1^ was determined. This means that the new pair of peaks has two linear pH-dependent regions, the first one involving two protons and two electrons, and the second one involving only one proton due to the deprotonation of this new electroactive group. This behavior is very similar to the well-known redox pair assigned to free FAD, the only small difference being the pKa value, which is higher in the first case.

Knowing that the number of electrons involved in the redox reaction is closer to two, a theoretical number of *n* = 2 was used to calculate the *α*_app_ and *k*_app_, following Equation (4) [53,56]. This peak at E_1/2_ = +0.155 V presents *α*_app_ = 0.40 and *k*_app_ = 1.38 s^−1^, which are close to the values reported for free FAD and summarized in [53]. Differences in the electron-transfer rate constants are related with the physico-chemical nature of the electrodes and the chemical nature of the group producing this faradaic activity [53,56,59].

#### 3.2.3. Evaluation of Glucose Detection

**Figure 7:**
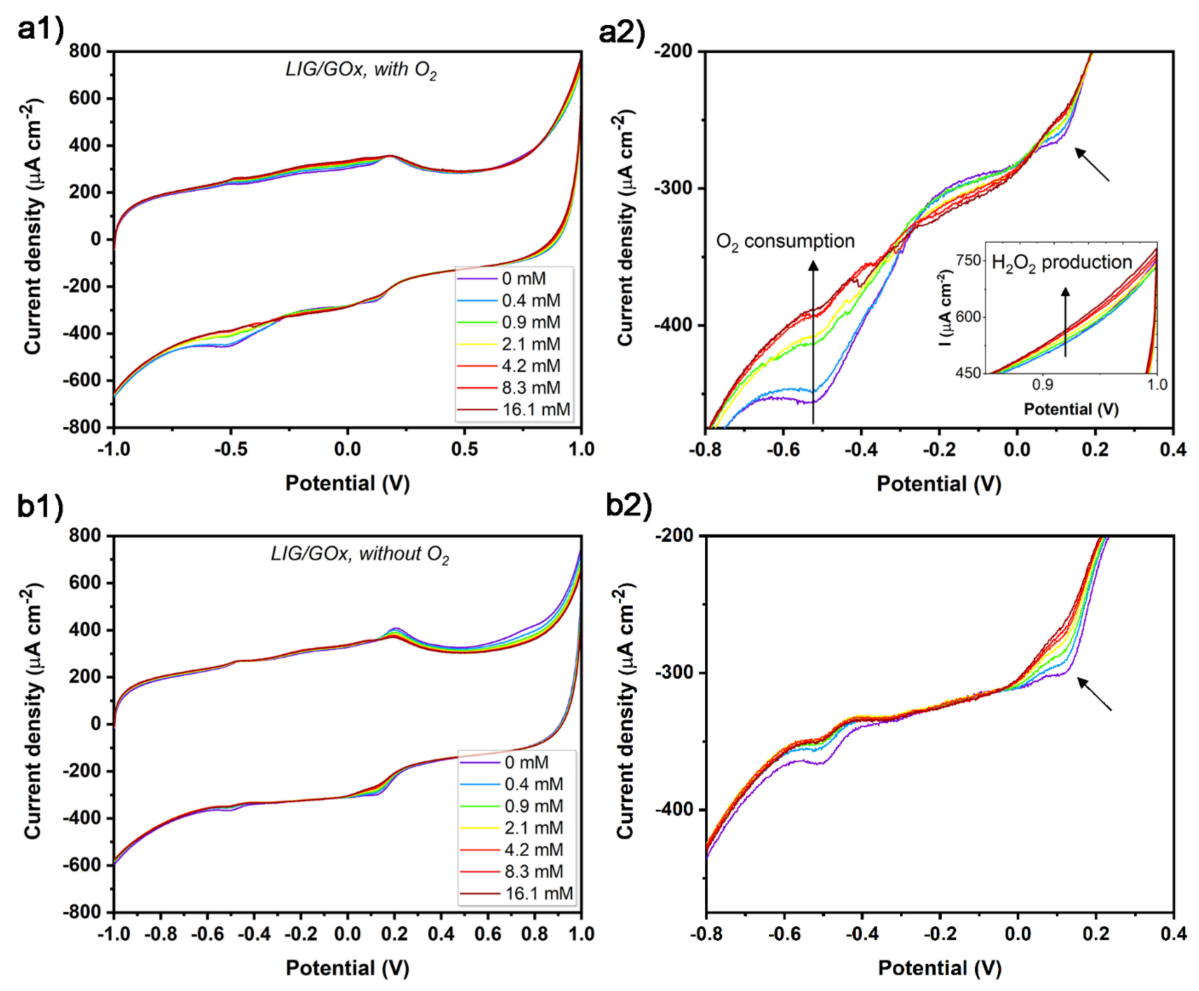
Cyclic voltammograms, from −1.0 to 1.0 V at 100 mV s^−1^, of LIG/GOx (**a1**) in the presence and (**b2**) in the absence of O_2_, varying glucose concentration. Three cycles were recorded and only the third is displayed. All the measurements were performed in PBS (pH 7.4, 10 mM). (**a2**) and (**b2**) are magnifications of the cathodic region of the voltammograms displayed in (**a1**) and (**b1**), respectively. The inset in (**a2**) presents the potential region where H_2_O_2_ electro-oxidation occurs.

LIG/GOx electrodes were further tested in the presence of glucose to understand if upon GOx adsorption on LIG the enzyme remains catalytically active even after the plug-into procedure, i.e., after run the CV up to +1.0 V. Here, mediator-free tests were chosen in order to also study the behavior of the new pair of peaks in the presence of glucose, without the interference of mediators that usually possess faradaic activity at such potentials [33,34,36]. To do so, small volumes of a glucose solution (500 mM) were added consecutively to the electrolyte, during stirring, and CVs were recorded after each addition, in the presence and absence of O_2_. The results are displayed in Figure 7.

In Figure 7a, glucose was added to the electrolyte solution in the presence of O_2_. As demonstrated in Figure 3 and Figure 4, and by other authors elsewhere [31], the reduction peak of free FAD overlapped with the reduction potential of O_2_. One must keep in mind that, in the past, this overlapping led to misleading interpretation of data that assigned this cathodic peak to the FAD in the active site of the enzyme. The overall observation of the voltammograms as a function of glucose concentrations, Figure 7a1, allows concluding that the capacitive currents were kept constant during glucose addition, and there is no appearance of new faradaic peaks. However, the peaks corresponding to electrochemical reduction of O_2_ between −0.6 and −0.4 V have changed in intensity (see the magnified Figure 7a2 and Equation (1)). Increasing the concentration of glucose, the current of this peak decreases as O_2_ is being consumed by GOx during glucose oxidation, according to Scheme 1. Hence, H_2_O_2_ is produced and electrochemically oxidized at circa +0.9 V following Equation (2), as seen in the inset of Figure 7a2. This result demonstrates that the adsorbed enzyme is still active. Indeed, it is important to note that LIG electrodes are able to detect H_2_O_2_ as presented and briefly discussed in Supplementary Materials, see Figure S7. Furthermore, the cathodic peak current at circa +0.114 V also decreases with increasing glucose concentration.

Regarding the glucose detection tests in the absence of O_2_, as depicted in Figure 7b1, no changes were expected in the cathodic peak of free FAD (−0.510 V), which is overlapped with the O_2_ reduction peak. However, a slight current decrease was observed (Figure 7b2), maybe due to residual O_2_ dissolved in the electrolyte, which was consumed by the enzyme in the first addition of glucose to the electrolyte. Concerning the cathodic peak at +0.114 V, also in O_2_ free conditions, the current has decreased by increasing the glucose concentration (Figure 7b2).

#### 3.2.4. Critical Analysis of GOx Electrochemistry on LIG

In this work, GOx was adsorbed on LIG electrodes and after running a CV from −0.2 to +0.6 V no faradaic activity was seen. However, running a CV up to +1.0 V, a new pair of peaks was observed with a E_1/2_ = +0.155 V vs. Ag/AgCl (1 M KCl). Moreover, a pair of peaks (E_1/2_ = −0.485 V vs. Ag/AgCl (1 M KCl)) corresponding to adsorbed free FAD was also noticed, as discussed elsewhere [31]. This procedure demonstrated that some electrooxidation reaction occurs between the LIG electrodes and GOx, giving rise to the appearance of the new pair of peaks. It is important to remember that, after several cycles from −1.0 to +1.0 V, neither the bare LIG nor the LIG/FAD electrodes presented such a pair of peaks. Therefore, it must be entirely related to LIG/GOx interaction. In addition, this pair of peaks is sensitive to the increase in glucose concentration and thus it must be related to electron transfer from GOx to the electrode’s surface. Hence, we propose two hypotheses: (i) the occurrence of DET or, (ii) that the plug-into procedure yields an in situ redox mediator allowing MET. Regarding the first hypothesis, FAD is buried in the active site of the enzyme, thus far from the electrode’s surface which makes the occurrence of the direct electron transfer difficult [25]. If some partial conformational change took place during the plug-into procedure, thus decreasing the distance between FAD and the electrode surface, DET could occur at +0.155 V vs. Ag/AgCl (1 M KCl). However, the redox potential of GOx in solution was determined by Vogt et al. 2014 [60], who measured by UV/vis spectroelectrochemistry a value of −0.385 V vs. Ag/AgCl (1 M KCl), at pH = 7.4. Since then, this value has been used as reference for the redox potential value of GOx. Concerning the second hypothesis, the plug-into procedure could promote the formation of a redox mediator by the reaction between amino acids in the entrance of the active site and functional groups of LIG, enabling mediated electron transfer from FAD in the active site to LIG surface. Indeed, in engineering procedures of GOx, redox mediators have been successfully bonded to GOx in order to enhance the electron transfer rate, as reviewed in [25].

The pH-dependence study confirms that these peaks arise from a two proton and two electron redox reaction, which is typical of quinone derivatives [61,62], of which FAD/FADH_2_ redox reaction is one example. Moreover, concerning the pH range where this reaction occurs, it varies depending on the pKa, however, the pKa of the free FAD also shifted to higher values after its adsorption on LIG due to their strong interaction, as also observed in other carbon-based electrodes [54], thus hampering the comparison with other quinone derivatives. In addition, cyclic voltammetry of LIG/GOx electrodes was carried out at room temperature (21 °C), 30 °C and 37 °C (Figure S6 in Supplementary Materials), showing that the new pair of peaks and the pair of peaks corresponding to free FAD seem to be related, as increasing the electrolyte solution temperature decreases the current of the new pair of peaks, whereas the FAD peaks’ currents increase, see in more detail Section 3.2 in Supplementary Materials.

If partial conformational changes took place in the enzyme, and DET was observed, was the enzyme still active to catalyze the oxidation of glucose? Indeed, catalytic oxidation of glucose was observed by the decrease in peak current corresponding to O_2_. This allows concluding that at least a percentage of the adsorbed enzyme is active. But is the enzyme performing DET active? On one hand, the presence of O_2_ results in a smaller decrease in the current compared with the absence of O_2_. Since the fully oxidized form of GOx, GOx(FAD), oxidizes glucose, according to Scheme 1a, the concentration of GOx(FAD) decreases by increasing the concentration of glucose and the reaction described by Equation (3) (direction 1) occurs to a lesser extent. When in the presence of O_2_, Scheme 1b does take place and GOx(FADH_2_) is regenerated. Consequently competition between both reactions takes place and a lower response in the cathodic peak of GOx(FAD) is observed. In the absence of O_2_, the resultant GOx(FADH_2_) is not regenerated. This scenario means that the enzyme with the partial conformational changes that enable DET is still catalytically active and able to oxidize glucose. Still, this hypothesis does not rule out a second hypothesis where a quinone-based mediator was created in the LIG/GOx interface without the loss of catalytic properties.

An additional experiment was performed using the cholesterol oxidase (ChOx) enzyme. In this case, the ChOx catalyzes the oxidation of cholesterol trough the FAD in its active site, which is also buried [63]. A similar pair of peaks was also observed after running the CV up to +1.0 V vs. Ag/AgCl (1 M KCl), see Figure S8 in Supplementary Materials. Another control test was conducted using GOx adsorbed on commercial screen-printed electrodes (SPE) of carbon modified with graphene oxide (GO), Figures S9 and S10. In this case, only the pair of peaks corresponding to adsorbed free FAD was observed. Even though, Alwarappan et al. 2012 [64], observed a well-defined pair of peaks with the reduction peak centered at +0.18 V vs. Ag/AgCl (3.0 M KCl), at pH 7.4, when GOx is conjugated with graphene sheets. Additionally, in the work of Wooten et al. 2014 [46] using CNTs electrodes, a weak pair of peaks (between +0.1 and +0.2 V) was observed when the authors ran the CV up to +0.8 V. Whilst this could also be ascribed to the same mechanism reported herein, no mention or discussion regarding this was reported.

## 4. Conclusions

In this work, FAD and GOx were adsorbed on LIG electrodes and an analysis of their electrochemical interaction was performed. Similar to other carbon materials, FAD strongly adsorbed on LIG and presented a half-wave potential at −0.490 V vs. Ag/AgCl (1 M). Concerning the LIG/GOx electrodes, two pairs of peaks were observed after running the CV up to +1.0 V vs. Ag/AgCl (1 M KCl), at pH 7.4, in the absence of O_2_. The first pair of peaks, with E_1/2_ = −0.485 V, is undoubtedly assigned to free FAD as an impurity of the GOx powder. The second one with E_1/2_ = +0.155 V vs. Ag/AgCl (1 M KCl) is more difficult to unequivocally ascribe. Still, several pieces of evidence are presented and discussed pointing out (i) the possibility of DET being observed for the first time using LIG electrodes, or (ii) the formation of a quinone derivative which mediates electron transfer from GOx to LIG. A plug-into procedure was required for this to happen, thus some chemical reaction occurred between the LIG electrode and GOx when the CV was run up to +1.0 V. Consequently, some partial conformational changes in the enzyme must have occurred and the FAD in the active site of the enzyme got closer to the electrode surface, allowing DET, or in the possibility of MET occurrence, this procedure yielded the in situ formation of a redox mediator. Moreover, the results indicate that glucose can be detected through this reaction. In-depth studies should be conducted in the future to get further insights into the definite origin of these peaks.

## Data Availability

The raw/processed data required to reproduce these findings can be shared upon request.

## Supplementary Materials

The following are available online at https://www.mdpi.com/article/10.3390/nano11081893/s1, Figure S1: (a and b) HR-TEM images of the bare LIG after synthesis. (b) is a high magnification of the area in the yel-low square in (a); Figure S2: Photograph of the home-made three-electrode configuration: LIG is the working electrode (WE), Ag/AgCl (1 M KCl) is the reference electrode (RE) and a Pt wire is the counter electrode (CE). There is an inlet for N_2_ allowing the purging of O_2_; Figure S3: Cyclic voltammograms (5 cycles) of LIG before and after immobilization of GOx, in a range potential from -0.2 to 0.6 V at 100 mV s^−1^. The measurements were performed in PBS (pH 7.4, 10 mM) after purging the electrolyte with N_2_ during 30 min to remove the O_2_. The potentials are measured against Ag/AgCl (1 M KCl); Figure S4: Cyclic voltammograms (5 cycles), from -0.2 to 0.6 V at 100 mV s^−1^, of LIG/GOx before and after the cyclic voltammetry measures from −0.2 to 1.0 V at 100 mV s^−1^. The measurements were performed in PBS (pH 7.4, 10 mM) after purging the electrolyte with N_2_ during 30 min to remove the O_2_. The potentials are measured against Ag/AgCl (1 M KCl); Figure S5: Cyclic voltammograms, from −1.0 to 1.0 V at 100 mV s^−1^, of one of the LIG electrodes functionalized with GOx, in the presence and absence of O_2_ in the electrolyte (PBS, pH = 7.4, 10 mM). Five cycles were performed and only the 5th cycle is displayed. The potentials were measured against Ag/AgCl (1 M KCl); Figure S6: Cyclic voltammograms, from −0.8 to 1.0 V at 100 mV s^−1^, of one electrode of LIG/GOx varying the temperature from 21 °C to 37 °C. Five cycles were recorded and only the 5th is displayed. All the measurements were performed in PBS (pH 7.4, 10 mM), in the absence of O_2_; Figure S7: (a) Cyclic voltammograms from −1.0 to 1.0 V at 100 mV s^−1^ using a LIG electrode. The measurements were performed in PBS (pH 7.4, 10 mM) after purging the electrolyte with N_2_ during 30 min to remove the O_2_. The potentials are measured against Ag/AgCl (1 M KCl). (b) Intensity of the current for each peak identified in (a) as function of the H2O2 concentration. A linear fitting was calculated for the peak corresponding to oxidation of H_2_O_2_ and reduction of O_2_; Figure S8: LIG and LIG/ChOx in a range potential from −0.8 to 1.0 V, at 100 mV s^−1^. All the measurements were performed in PBS (pH 7.4, 10 mM) in the absence of O_2_ and the potentials are measured against Ag/AgCl (1 M KCl). Note that this LIG/ChOx CV was recorded after the plug-into CVs; Figure S9: Cyclic voltammograms, from −1.0 to 1.0 V at 100 mV s^−1^, of a SPE of carbon modified with graphene oxide. Performing 15 cycles (3 × 5cycles) were needed to stabilize the electrode before being used. PBS (pH 7.4, 10 mM) was used as electrolyte and the potentials were measured against Ag/AgCl (1 M KCl); Figure S10. Cyclic voltammograms, from −1.0 to 1.0 V at 100 mV s^−1^, of SPE of carbon modified with graphene oxide, after the stabilization procedure as represented in Figure S9. In total, 10 cycles were performed (2 × 5 cycles) for each electrode. In (a) CV of the electrode after being incubated with a drop of PBS (100 μL) overnight; and (b) after being incubated with a drop of GOx (100 μL, 5 mg mL^−1^ in PBS) overnight. PBS (pH 7.4, 10 mM) was used as electrolyte, and it was bubbled with N2 during 30 min to remove the O_2_ in solution before CVs acquisition. The potentials were measured against Ag/AgCl (1 M KCl).

## References

[1] Tiwari J.N., Vij V., Kemp K.C., Kim K.S. (2016). Engineered Carbon-Nanomaterial-Based Electrochemical Sensors for Biomolecules. ACS Nano.

[2] Wang W., Su H., Wu Y., Zhou T., Li T. (2019). Review—Biosensing and Biomedical Applications of Graphene: A Review of Current Progress and Future Prospect. J. Electrochem. Soc..

[3] Lawal A.T. (2018). Progress in utilisation of graphene for electrochemical biosensors. Biosens. Bioelectron..

[4] Santos N.F., Pereira S.O., Moreira A., Girão A.V., Carvalho A.F., Fernandes A.J.S., Costa F.M. (2021). IR and UV Laser-Induced Graphene: Application as Dopamine Electrochemical Sensors. Adv. Mater. Technol..

[5] Ambrosi A., Chua C.K., Bonanni A., Pumera M. (2014). Electrochemistry of graphene and related materials. Chem. Rev..

[6] Huang X., Yin Z., Wu S., Qi X., He Q., Zhang Q., Yan Q., Boey F., Zhang H. (2011). Graphene-based materials: Synthesis, characterization, properties, and applications. Small.

[7] Lee J.-H., Park S., Choi J.-W. (2019). Electrical Property of Graphene and Its Application to Electrochemical Biosensing. Nanomaterials.

[8] Lu L. (2018). Recent advances in synthesis of three-dimensional porous graphene and its applications in construction of electrochemical (bio)sensors for small biomolecules detection. Biosens. Bioelectron..

[9] Lin J., Peng Z., Liu Y., Ruiz-Zepeda F., Ye R., Samuel E.L.G., Yacaman M.J., Yakobson B.I., Tour J.M. (2014). Laser-induced porous graphene films from commercial polymers. Nat. Commun..

[10] Ye R., James D.K., Tour J.M. (2019). Laser-Induced Graphene: From Discovery to Translation. Adv. Mater..

[11] Carvalho A.F., Fernandes A.J.S., Leitão C., Deuermeier J., Marques A.C., Martins R., Fortunato E., Costa F.M. (2018). Laser-Induced Graphene Strain Sensors Produced by Ultraviolet Irradiation of Polyimide. Adv. Funct. Mater..

[12] Gao J., He S., Nag A. (2021). Electrochemical Detection of Glucose Molecules Using Laser-Induced Graphene Sensors: A Review. Sensors.

[13] Lahcen A.A., Rauf S., Beduk T., Durmus C., Aljedaibi A., Timur S., Alshareef H.N., Amine A., Wolfbeis O.S., Salama K.N. (2020). Electrochemical sensors and biosensors using laser-derived graphene: A comprehensive review. Biosens. Bioelectron..

[14] Tehrani F., Reiner L., Bavarian B. (2015). Rapid Prototyping of a High Sensitivity Graphene Based Glucose Sensor Strip. PLoS ONE.

[15] Tehrani F., Bavarian B. (2016). Facile and scalable disposable sensor based on laser engraved graphene for electrochemical detection of glucose. Sci. Rep..

[16] Yoon H., Nah J., Kim H., Ko S., Sharifuzzaman M., Barman S.C., Xuan X., Kim J., Park J.Y. (2020). A chemically modified laser-induced porous graphene based flexible and ultrasensitive electrochemical biosensor for sweat glucose detection. Sens. Actuators B Chem..

[17] Lu Z., Wu L., Dai X., Wang Y., Sun M., Zhou C., Du H., Rao H. (2021). Novel flexible bifunctional amperometric biosensor based on laser engraved porous graphene array electrodes: Highly sensitive electrochemical determination of hydrogen peroxide and glucose. J. Hazard. Mater..

[18] Bauer M., Wunderlich L., Weinzierl F., Lei Y., Duerkop A., Alshareef H.N., Baeumner A.J. (2021). Electrochemical multi-analyte point-of-care perspiration sensors using on-chip three-dimensional graphene electrodes. Anal. Bioanal. Chem..

[19] Nayak P., Kurra N., Xia C., Alshareef H.N. (2016). Highly Efficient Laser Scribed Graphene Electrodes for On-Chip Electrochemical Sensing Applications. Adv. Electron. Mater..

[20] Xu G., Jarjes Z.A., Desprez V., Kilmartin P.A., Travas-Sejdic J. (2018). Sensitive, selective, disposable electrochemical dopamine sensor based on PEDOT-modified laser scribed graphene. Biosens. Bioelectron..

[21] Fenzl C., Nayak P., Hirsch T., Wolfbeis O.S., Alshareef H.N., Baeumner A.J. (2017). Laser-Scribed Graphene Electrodes for Aptamer-Based Biosensing. ACS Sens..

[22] Mamleyev E.R., Heissler S., Nefedov A., Weidler P.G., Nordin N., Kudryashov V.V., Länge K., MacKinnon N., Sharma S. (2019). Laser-induced hierarchical carbon patterns on polyimide substrates for flexible urea sensors. NPJ Flex. Electron..

[23] Cardoso A.R., Marques A.C., Santos L., Carvalho A.F., Costa F.M., Martins R., Sales M.G.F., Fortunato E. (2019). Molecularly-imprinted chloramphenicol sensor with laser-induced graphene electrodes. Biosens. Bioelectron..

[24] Marques A.C., Cardoso A.R., Martins R., Sales M.G.F., Fortunato E. (2020). Laser-Induced Graphene-Based Platforms for Dual Biorecognition of Molecules. ACS Appl. Nano Mater..

[25] Mano N. (2019). Engineering glucose oxidase for bioelectrochemical applications. Bioelectrochemistry.

[26] Sehit E., Altintas Z. (2020). Significance of nanomaterials in electrochemical glucose sensors: An updated review (2016–2020). Biosens. Bioelectron..

[27] Teymourian H., Barfidokht A., Wang J. (2020). Electrochemical glucose sensors in diabetes management: An updated review (2010–2020). Chem. Soc. Rev..

[28] Clark L.C., Lyons C. (1962). Electrode systems for continuous monitoring in cardiovascular surgery. Ann. N. Y. Acad. Sci..

[29] Hecht H.J., Kalisz H.M., Hendle J., Schmid R.D., Schomburg D. (1993). Crystal Structure of Glucose Oxidase from Aspergillus niger Refined at 2·3 Å Reslution. J. Mol. Biol..

[30] Hecht H.J., Schomburg D., Kalisz H., Schmid R.D. (1993). The 3D structure of glucose oxidase from Aspergillus niger. Implications for the use of GOD as a biosensor enzyme. Biosens. Bioelectron..

[31] Bartlett P.N., Al-Lolage F.A. (2018). There is no evidence to support literature claims of direct electron transfer (DET) for native glucose oxidase (GOx) at carbon nanotubes or graphene. J. Electroanal. Chem..

[32] Leskovac V., Trivić S., Wohlfahrt G., Kandrač J., Peričin D. (2005). Glucose oxidase from Aspergillus niger: The mechanism of action with molecular oxygen, quinones, and one-electron acceptors. Int. J. Biochem. Cell Biol..

[33] Cass A.E.G., Davis G., Francis G.D., Hill H.A.O., Aston W.J., Higgins I.J., Plotkin E.V., Scott L.D.L., Turner A.P.F. (1984). Ferrocene-mediated enzyme electrode for amperometric determination of glucose. Anal. Chem..

[34] Wang Y., Yao Y. (2012). Direct electron transfer of glucose oxidase promoted by carbon nanotubes is without value in certain mediator-free applications. Microchim. Acta.

[35] Poletti F., Favaretto L., Kovtun A., Treossi E., Corticelli F., Gazzano M., Palermo V., Zanardi C., Melucci M. (2020). Electrochemical sensing of glucose by chitosan modified graphene oxide. J. Phys. Mater..

[36] Goran J.M., Mantilla S.M., Stevenson K.J. (2013). Influence of Surface Adsorption on the Interfacial Electron Transfer of Flavin Adenine Dinucleotide and Glucose Oxidase at Carbon Nanotube and Nitrogen-Doped Carbon Nanotube Electrodes. Anal. Chem..

[37] Murthy A.S.N., Sharma J. (1997). Benzoquinone-mediated enzyme biosensor for amperometric determination of glucose. Proc. Indian Acad. Sci. (Chem. Sci.).

[38] Zayats M., Katz E., Willner I. (2002). Electrical Contacting of Glucose Oxidase by Surface-Reconstitution of the Apo-Protein on a Relay-Boronic Acid-FAD Cofactor Monolayer. J. Am. Chem. Soc..

[39] Zheng L., Li J., Xu J., Xiong L., Zheng D., Liu Q., Liu W., Li Y., Yang S., Xia J. (2010). Improvement of amperometric glucose biosensor by the immobilization of FcCD inclusive complex and carbon nanotube. Analyst.

[40] Xiao Y., Patolsky F., Katz E., Hainfeld J.F., Willner I. (2003). “Plugging into Enzymes”: Nanowiring of Redox Enzymes by a Gold Nanoparticle. Science (80-).

[41] Holland J.T., Lau C., Brozik S., Atanassov P., Banta S. (2011). Engineering of Glucose Oxidase for Direct Electron Transfer via Site-Specific Gold Nanoparticle Conjugation. J. Am. Chem. Soc..

[42] Ban K., Ueki T., Tamada Y., Saito T., Imabayashi S., Watanabe M. (2001). Fast electron transfer between glucose oxidase and electrodes via phenothiazine mediators with poly(ethylene oxide) spacers attached to the enzyme surface. Electrochem. Commun..

[43] Suzuki N., Lee J., Loew N., Takahashi-Inose Y., Okuda-Shimazaki J., Kojima K., Mori K., Tsugawa W., Sode K. (2020). Engineered Glucose Oxidase Capable of Quasi-Direct Electron Transfer after a Quick-and-Easy Modification with a Mediator. Int. J. Mol. Sci..

[44] Krikstopaitis K., Kulys J., Tetianec L. (2004). Bioelectrocatalytical glucose oxidation with phenoxazine modified glucose oxidase. Electrochem. Commun..

[45] Bartlett P.N., Booth S., Caruana D.J., Kilburn J.D., Santamaría C. (1997). Modification of Glucose Oxidase by the Covalent Attachment of a Tetrathiafulvalene Derivative. Anal. Chem..

[46] Wooten M., Karra S., Zhang M., Gorski W. (2014). On the Direct Electron Transfer, Sensing, and Enzyme Activity in the Glucose Oxidase/Carbon Nanotubes System. Anal. Chem..

[47] Wilson G.S. (2016). Native glucose oxidase does not undergo direct electron transfer. Biosens. Bioelectron..

[48] Ferrari A.C., Basko D.M. (2013). Raman spectroscopy as a versatile tool for studying the properties of graphene. Nat. Nanotechnol..

[49] Eckmann A., Felten A., Mishchenko A., Britnell L., Krupke R., Novoselov K.S., Casiraghi C. (2012). Probing the Nature of Defects in Graphene by Raman Spectroscopy. Nano Lett..

[50] Rao R., Podila R., Tsuchikawa R., Katoch J., Tishler D., Rao A.M., Ishigami M. (2011). Effects of Layer Stacking on the Combination Raman Modes in Graphene. ACS Nano.

[51] Wang Y., Shao Y., Matson D.W., Li J., Lin Y. (2010). Nitrogen-Doped Graphene and Its Application in Electrochemical Biosensing. ACS Nano.

[52] Gorton L., Johansson G. (1980). Cyclic voltammetry of FAD adsorbed on graphite, glassy carbon, platinum and gold electrodes. J. Electroanal. Chem. Interfacial Electrochem..

[53] Wei H., Omanovic S. (2008). Interaction of Flavin Adenine Dinucleotide (FAD) with a Glassy Carbon Electrode Surface. Chem. Biodivers..

[54] Goran J.M., Stevenson K.J. (2013). Electrochemical Behavior of Flavin Adenine Dinucleotide Adsorbed onto Carbon Nanotube and Nitrogen-Doped Carbon Nanotube Electrodes. Langmuir.

[55] Kamal M.M., Elzanowska H., Gaur M., Kim D., Birss V.I. (1991). Electrochemistry of adsorbed flavin adenine dinucleotide in acidic solutions. J. Electroanal. Chem. Interfacial Electrochem..

[56] Shinohara H., Grätzel M., Vlachopoulos N., Aizawa M. (1991). Interfacial electron transfer of flavin coenzymes and riboflavin adsorbed on textured TiO2 films. J. Electroanal. Chem. Interfacial Electrochem..

[57] Liang B., Guo X., Fang L., Hu Y., Yang G., Zhu Q., Wei J., Ye X. (2015). Study of direct electron transfer and enzyme activity of glucose oxidase on graphene surface. Electrochem. Commun..

[58] Ksenzhek O.S., Petrova S.A. (1983). Electrochemical properties of flavins in aqueous solutions. Bioelectrochem. Bioenerg..

[59] Liu J., Paddon-Row M.N., Gooding J.J. (2004). Heterogeneous Electron-Transfer Kinetics for Flavin Adenine Dinucleotide and Ferrocene through Alkanethiol Mixed Monolayers on Gold Electrodes. J. Phys. Chem. B.

[60] Vogt S., Schneider M., Schäfer-Eberwein H., Nöll G. (2014). Determination of the pH Dependent Redox Potential of Glucose Oxidase by Spectroelectrochemistry. Anal. Chem..

[61] Blanchard P., Buzzetti P.H.M., Davies B., Nedellec Y., Girotto E.M., Gross A.J., Le Goff A., Nishina Y., Cosnier S., Holzinger M. (2019). Electrosynthesis of Pyrenediones on Carbon Nanotube Electrodes for Efficient Electron Transfer with FAD-dependent Glucose Dehydrogenase in Biofuel Cell Anodes. ChemElectroChem.

[62] Cobb S.J., Ayres Z.J., Newton M.E., Macpherson J.V. (2019). Deconvoluting Surface-Bound Quinone Proton Coupled Electron Transfer in Unbuffered Solutions: Toward a Universal Voltammetric pH Electrode. J. Am. Chem. Soc..

[63] Yue Q.K., Kass I.J., Sampson N.S., Vrielink A. (1999). Crystal Structure Determination of Cholesterol Oxidase from Streptomyces and Structural Characterization of Key Active Site Mutants †, ‡. Biochemistry.

[64] Alwarappan S., Singh S.R., Pillai S., Kumar A., Mohapatra S. (2012). Direct Electrochemistry of Glucose Oxidase at a Gold Electrode Modified with Graphene Nanosheets. Anal. Lett..

