# Supplementary material for: Electrochemical Response of Glucose Oxidase Adsorbed on Laser-Induced Graphene
Source: Nanomaterials (Basel). 2021 Jul 23;11(8):1893. doi: 10.3390/nano11081893 (PMC8401569; doi:10.3390/nano11081893)
Supplement: Supplementary file 1 [file nanomaterials-11-01893-s001.zip › nanomaterials-1283659-supplementary.pdf]

nanomaterials

Supplementary Information

Electrochemical Response of Glucose Oxidase Adsorbed on Laser-Induced Graphene

Sónia O. Pereira *, Nuno F. Santos, Alexandre F. Carvalho, António J. S. Fernandes and Florinda M. Costa

i3N, Department of Physics, University of Aveiro, 3810-193 Aveiro, Portugal; nfsantos@ua.pt (N.F.S.); alexandre.carvalho@ua.pt (A.F.C.); toze2@ua.pt (A.J.S.F.); flor@ua.pt (F.M.C.)
* Correspondence: sonia.pereira@ua.pt

1. HR-TEM of Bare LIG

LIG was removed from Kapton® and dispersed in ethanol for subsequent deposition in a carbon grid to be analyzed by HR-TEM. The observed microstructure is transparent to the electron beam and the sheets present different layer thickness, with a range from monolayer up to 8–10 layers, as shown in Figure S1. Furthermore, a d-spacing of 3.6 Å as measured which is characteristic of graphene hexagonal plane (111).

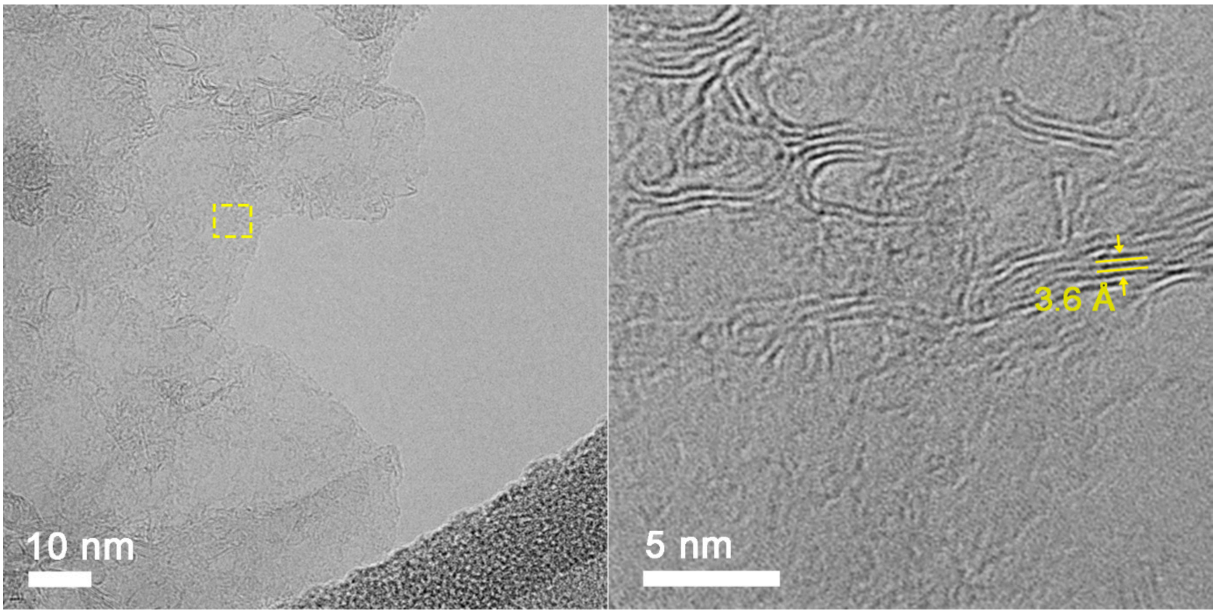

Figure S1. (**a** and **b**) HR-TEM images of the bare LIG after synthesis. (**b**) is a high magnification of the area in the yellow square in (**a**).

2. Electrochemical Setup

The electrochemical measurements were performed in a home-made three-electrode configuration setup, as displayed in Figure S2**.** The electrochemical cell has an inlet of N_2, which is bubbled inside of the electrolyte, at the bottom of the container, and an outlet for O_2 purging, in order to allow the performance of the electrochemical analysis under absence of O_2. Additionally, there is a small aperture with a lid, in the top of the container, allowing the addition of the glucose or H_2O_2, while in the absence of O_2.

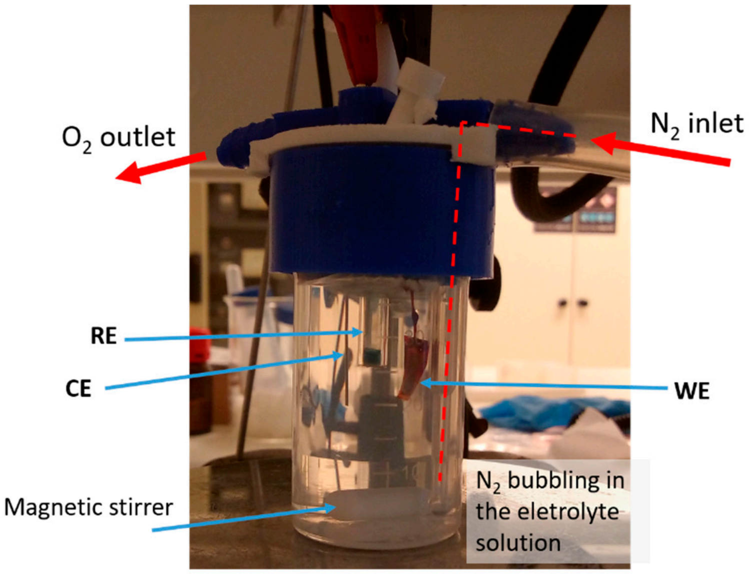

Figure S2. Photograph of the home-made three-electrode configuration: LIG is the working electrode (WE), Ag/AgCl (1 M KCl) is the reference electrode (RE) and a Pt wire is the counter electrode (CE). There is an inlet for N_2 allowing the purging of O_2.

3. Complementary Electrochemical Data

3.1. LIG Functionalized with Glucose Oxidase (GOx)

GOx was adsorbed on LIG electrodes and then electrochemical measurements were conducted. First a CV was performed in a potential range from −0.2 to 0.6 V, at 100 mV s^{-1}, but no peak was observed (Figure S3). After running CV from –0.2 to 1.0 V (or from –1.0 to 1.0 V, the same result was observed) a pair of redox peaks appeared, as displayed and discussed in the main manuscript concerning Figure 4. After this procedure, this pair of peaks always appear even when CV is run from –0.2 to 0.6 V (see Figure S4).

In addition, CV of GOx adsorbed on LIG electrodes was performed in the absence and presence of O_2, as depicted in Figure S5. Likewise to FAD CV in the absence and presence of O_2, the peaks shift to the left in the presence of O_2 and an increase of current in the cathodic peak is observed from –0.3 to –0.8 V due to the overlapped with the reduction potential of O_2 (see also Figure 3 in the main manuscript).

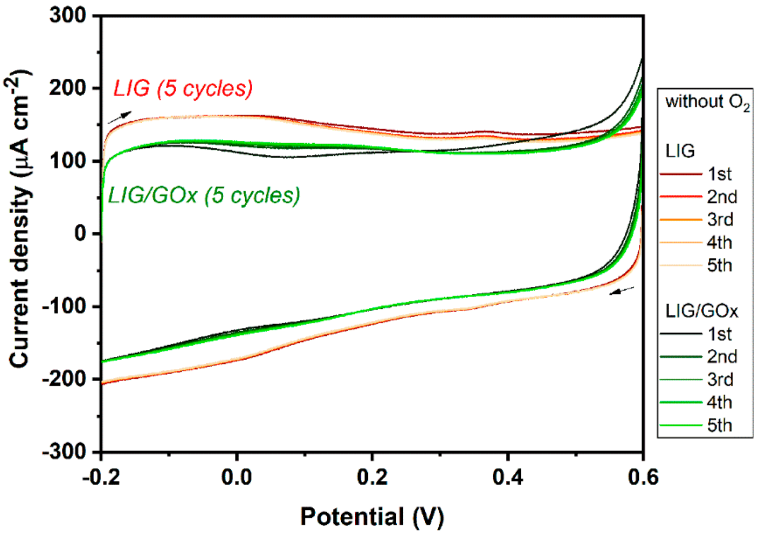

Figure S3. Cyclic voltammograms (5 cycles) of LIG before and after immobilization of GOx, in a range potential from -0.2 to 0.6 V at 100 mV s^{-1}. The measurements were performed in PBS (pH 7.4, 10 mM) after purging the electrolyte with N_2 during 30 min to remove the O_2. The potentials are measured against Ag/AgCl (1 M KCl).

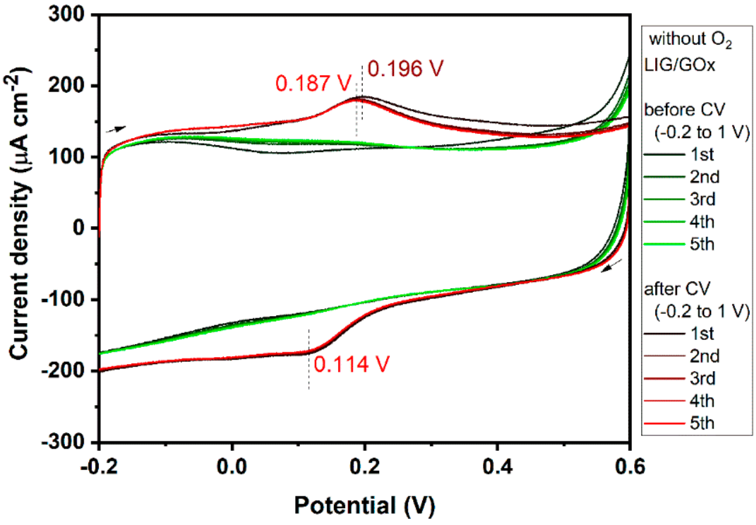

Figure S4. Cyclic voltammograms (5 cycles), from -0.2 to 0.6 V at 100 mV s-1, of LIG/GOx before and after the cyclic voltammetry measures from -0.2 to 1.0 V at 100 mV s-1. The measurements were performed in PBS (pH 7.4, 10 mM) after purging the electrolyte with N_2 during 30 min to remove the O_2. The potentials are measured against Ag/AgCl (1 M KCl).

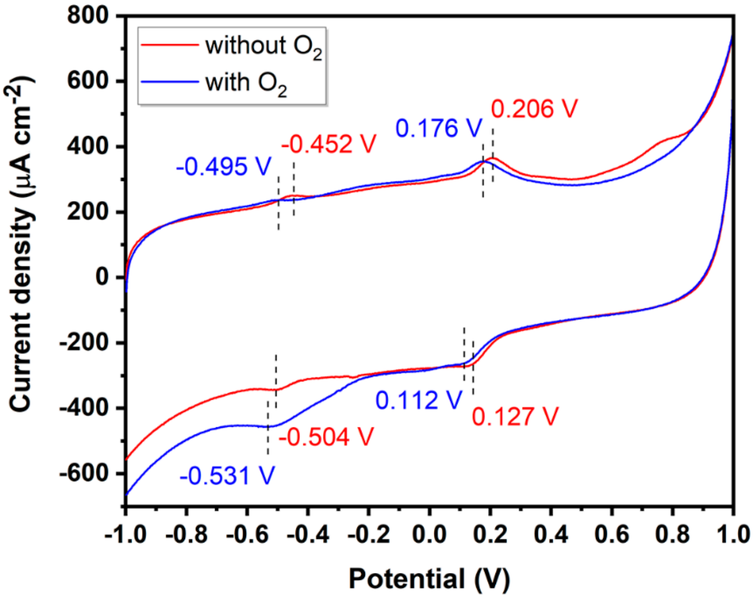

Figure S5. Cyclic voltammograms, from −1.0 to 1.0 V at 100 mV s-1, of one of the LIG electrodes functionalized with GOx, in the presence and absence of O_2 in the electrolyte (PBS, pH = 7.4, 10 mM). Five cycles were performed and only the 5th cycle is displayed. The potentials were measured against Ag/AgCl (1 M KCl).

3.2. Temperature-Dependence Study

All the measurements were performed in PBS (pH 7.4, 10 mM) as the electrolyte. The electrochemical cell was placed in a water bath at room temperature of 21 °C. The first CVs were acquired and then, under stirring, the temperature was increase to 30 °C, and afterwards to 37 °C.

In Figure S6, CV measurements are shown corresponding to three different electrolyte temperatures: 21 °C, 30 °C, and 37 °C. This study showed that increasing the electrolyte solution temperature, the new anodic and cathodic peaks current decrease. Furthermore, a shoulder in these peaks, at lower potentials, starts to be noticed with the increase in temperature, this behavior could be related with conformational changes of the enzyme, which shifts to lower potentials the electroactivity of the functional group responsible for this. Concerning the cathodic peak current, the precise evaluation of its change is hindered by the background currents increase at more negative potentials. This behavior indicates that both pairs of peaks seem to be related.

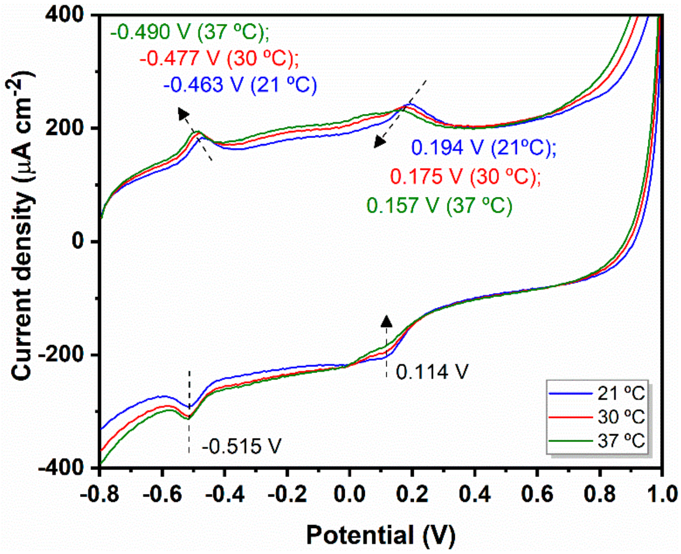

Figure S6. Cyclic voltammograms, from −0.8 to 1.0 V at 100 mVs^{-1}, of one electrode of LIG/GOx varying the temperature from 21 °C to 37 °C. Five cycles were recorded and only the 5th is displayed. All the measurements were performed in PBS (pH 7.4, 10 mM), in the absence of O_2.

3.3. LIG as a Transducer for H_2O_2 Detection

LIG electrodes were tested as transducers for the detection of H_2O_2, since this is a product of glucose enzymatic oxidation when in the presence of O_2. Similarly, prior to use the LIG electrodes were submitted to the conditioning procedure in order to stabilize the electrochemical background. Then, the electrolyte, PBS (pH 7.4, 10 mM), was bubbled with N_2 during 30 min in order to remove the O_2 and 5 cycles of CV were recorded. Afterwards, under stirring and keeping a gentle flux of N_2 guaranteeing the O_2 absence, small volumes of H_2O_2 3% (30, 60, 150, 300, 600, and 1200 µL) were added, to an initial electrolyte volume of 35 mL. After each volume addition, 3 CV were run, but only the 3rd cycle is displayed in Figure S7.

The oxidation of H_2O_2 occurs at circa +0.9 V vs Ag/AgCl (1 M KCl), as reported elsewhere for carbon based electrodes [1], following Equation S2**.** During H_2O_2 oxidation, O2 is generated and subsequently it is electrochemically reduced as observed by the current increase between –0.6 and –0.4 V. Both reactions present a linear response up to 7.4 mM of H_2O_2. (see Figure S7). After that a saturation seems to occur and a third peak appears for the higher H_2O_2 concentrations. This peak is assignable to the oxidation of O_2. Note that LIG is a very porous material produced in normal atmospheric conditions and the pores are filled with air.

$$O_2 + 4H^+ + 4e^- \longrightarrow 2H_2O \quad \textbf{(S1)}$$

$$H_2O_2 \longrightarrow 2H^+ + 2e^- + O_2 \quad \textbf{(S2)}$$

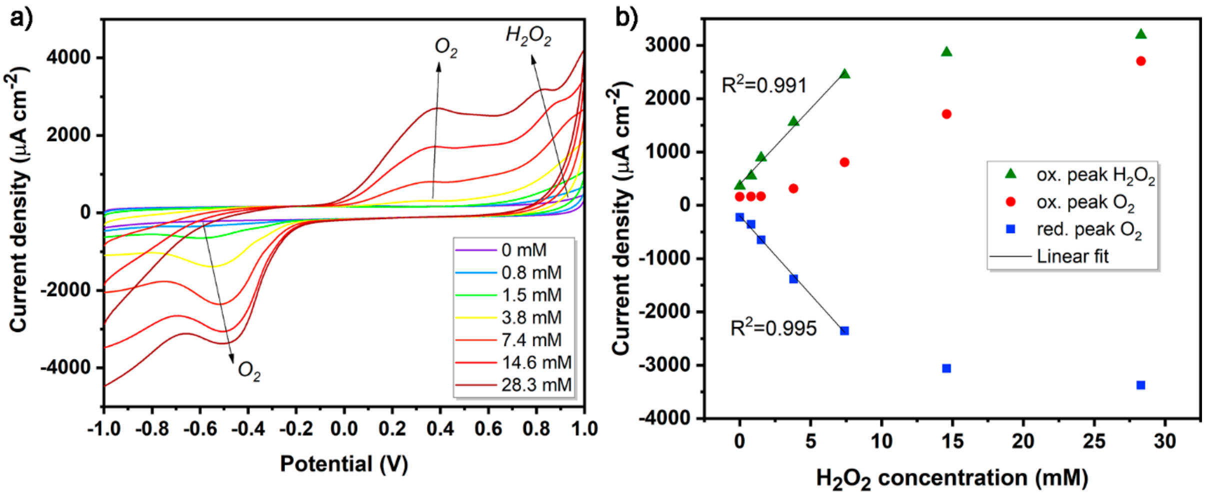

Figure S7. (**a**) Cyclic voltammograms from –1.0 to 1.0 V at 100 mV s^{-1} using a LIG electrode. The measurements were performed in PBS (pH 7.4, 10 mM) after purging the electrolyte with N_2 during 30 min to remove the O_2. The potentials are measured against Ag/AgCl (1 M KCl). (**b**) Intensity of the current for each peak identified in (**a**) as function of the H_2O_2 concentration. A linear fitting was calculated for the peak corresponding to oxidation of H_2O_2 and reduction of O_2.

3.4. Cholesterol Oxidase (ChOx) Adsorbed on LIG Electrodes

First, the LIG electrode was submitted to the conditioning procedure in order to stabilize the electrochemical background. Then the electrolyte was bubbled during 30 min to remove the O_2 and 5 cycles of CV were recorded, see Figure S8.

Second, Cholesterol oxidase was immobilized on LIG electrode by physical adsorption. The LIG electrode was immersed in a fresh solution of the enzyme (0.5 mg mL^{-1}) overnight, and it as kept at 10 °C. Afterwards, the electrode was exhaustively washed with PBS to remove non-adsorbed enzyme. The LIG/ChOx electrode was placed in the electrolyte, the O_2 was removed and the "plug-in" CV were run. A further 5 cycles were performed, as displayed in Figure S8.

Similarly to GOx, and as discussed in the main manuscript, a pair of peaks corresponding to free FAD and ChOx(FAD) were observed at a half-wave potential of -0.483 V and 0.155 V (vs Ag/AgCl, 1 M KCl), respectively.

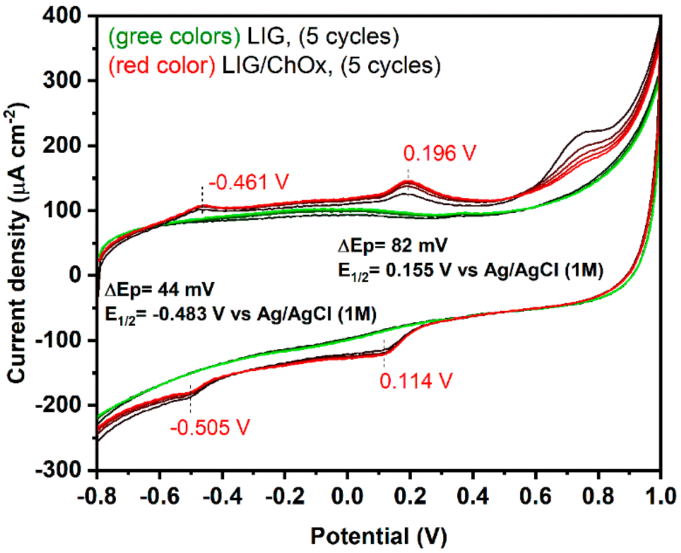

Figure S8. LIG and LIG/ChOx in a range potential from -0.8 to 1.0 V, at 100 mV s^{-1}. All the measurements were performed in PBS (pH 7.4, 10 mM) in the absence of O_2 and the potentials are measured against Ag/AgCl (1 M KCl). Note that this LIG/ChOx CV was recorded after the plug-into CVs.

3.5. Screen-Printed Electrodes (SPE)

The screen-printed electrodes (SPE) of carbon modified with graphene oxide (110GPHOX) were purchased from Metrohm DropSens (Oviedo, Spain). In the case of the SPE, the electrode contains the working electrode (WE) of graphene oxide/carbon (geometric area of 0.11 cm^2) and silver and carbon as reference and counter electrodes (RE and CE), respectively. However, in order to create a possible comparison with LIG electrodes, the RE and CE were isolated using an insulating varnish (Lancomit from Agar Scientific) and an electrical contact was established using a copper wire and silver ink (Electrodag 1415 from Agar Scientific) baked at 120 °C for 20 min. The electrical contact was also covered with the insulating varnish and the same home-made three-electrode configuration set up, as well as the same electrochemical station were used, as described in the manuscript. Just to clarify, Ag/AgCl (1 M KCl) (CHI111, CH Instruments, Inc) was used as the RE and a Pt wire as the CE. All the measurements were performed in 35 mL of PBS (pH 7.4, 10 mM) as electrolyte at room temperature.

3.5.1. SPE Electrochemical Stabilization

Similarly to LIG, SPE was submitted to a conditioning procedure in order to stabilize the electrochemical response before being used throughout the work. In the case of SPE, all the electrodes were also immersed in PBS circa 30 min, but for the electrochemical stabilization procedure 15 cycles (3 × 5 cycles) of voltammograms were required from –1.0 to 1.0 V at 100 mV s^{-1}, using PBS (pH 7.4, 10 mM) as electrolyte. See the voltammograms in Figure S9.

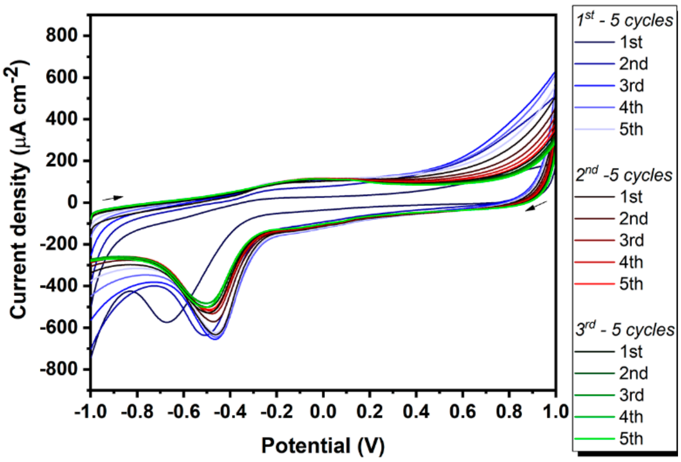

Figure S9. Cyclic voltammograms, from -1.0 to 1.0 V at 100 mV s^{-1}, of a SPE of carbon modified with graphene oxide. Performing 15 cycles (3 × 5cycles) were needed to stabilize the electrode before being used. PBS (pH 7.4, 10 mM) was used as electrolyte and the potentials were measured against Ag/AgCl (1 M KCl).

3.5.2. SPE Functionalization

After the stabilization procedure, a drop containing GOx (100 μL, 5 mg mL^{-1} in PBS) was placed in two electrodes during the night (circa 16h) without drying, keeping them in a humid atmosphere in a refrigerated box. Afterwards the electrodes were extensively washed with PBS before the electrochemical measurements. For comparison purposes, a reference electrode was also prepared, but the 100 μL drop was only the solution of PBS. See the resulting voltammograms in Figure S10.

Note that the presence of the enzyme increases the capacitance of the electrode and a pair of peaks is observed with a formal potential of –0.491 V, which is assigned to free FAD adsorbed onto the electrode, similar to the results find for LIG electrodes. In addition the first cycle in (b), corresponding to the SPE functionalized with GOx, have a similar

behavior as LIG/GOx. However, unlike LIG/GOx, during the performance of the cycles no additional peaks are noticeable, as discussed in the main manuscript.

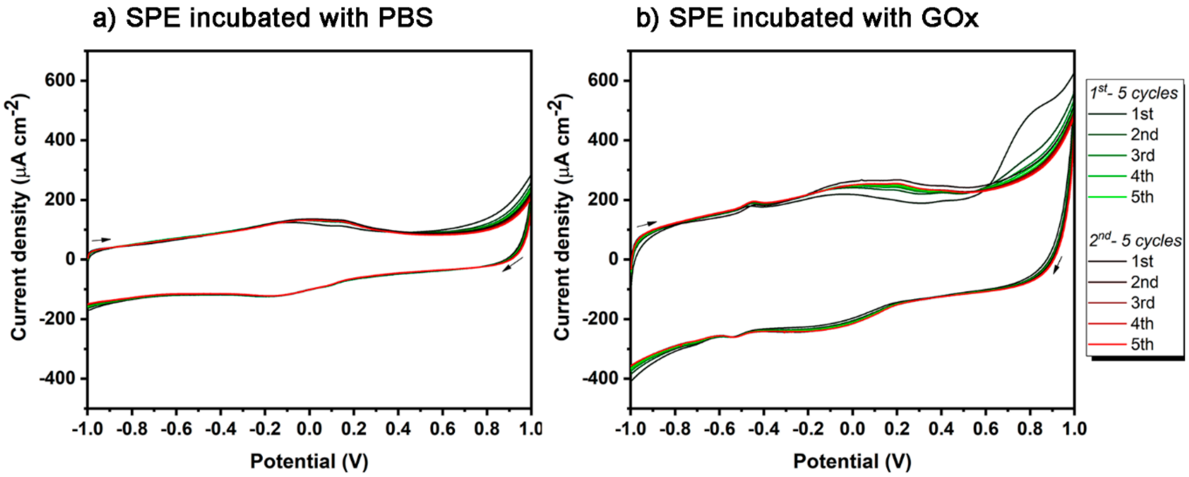

Figure S10. Cyclic voltammograms, from –1.0 to 1.0 V at 100 mV s^{-1}, of SPE of carbon modified with graphene oxide, after the stabilization procedure as represented in Figure S9. In total, 10 cycles were performed (2 × 5 cycles) for each electrode. In (**a**) CV of the electrode after being incubated with a drop of PBS (100 μL) overnight; and (**b**) after being incubated with a drop of GOx (100 μL, 5mg mL^{-1} in PBS) overnight. PBS (pH 7.4, 10 mM) was used as electrolyte and it was bubbled with N_2 during 30 min to remove the O_2 in solution before CVs acquisition. The potentials were measured against Ag/AgCl (1 M KCl).

References

1. Wooten, M.; Karra, S.; Zhang, M.; Gorski, W. On the Direct Electron Transfer, Sensing, and Enzyme Activity in the Glucose Oxidase/Carbon Nanotubes System. *Anal. Chem.* **2014**, *86*, 752–757, doi:10.1021/ac403250w.
